# Remarkable Cryptic Diversity of *Paratylenchus* spp. (Nematoda: Tylenchulidae) in Spain †

**DOI:** 10.3390/ani11041161

**Published:** 2021-04-18

**Authors:** Ilenia Clavero-Camacho, Carolina Cantalapiedra-Navarrete, Antonio Archidona-Yuste, Pablo Castillo, Juan Emilio Palomares-Rius

**Affiliations:** 1 Campus de Excelencia Internacional Agroalimentario, ceiA3, Instituto de Agricultura Sostenible (IAS), Consejo Superior de Investigaciones Científicas (CSIC), Avenida Menéndez Pidal s/n, 14004 Córdoba, Spain; iclavero@ias.csic.es (I.C.-C.); ccantalapiedra@ias.csic.es (C.C.-N.); p.castillo@csic.es (P.C.); 2 Centro Alameda del Obispo, Andalusian Institute of Agricultural and Fisheries Research and Training (IFAPA), 14004 Córdoba, Spain; antonio.archidona-yuste@ufz.de; 3 Helmholtz Centre for Environmental Research-FZ, Department of Ecological Modelling, Permoserstrasse 15, 04318 Leipzig, Germany

**Keywords:** cytochrome c oxidase subunit 1, ITS rRNA, D2-D3 of 28S rRNA, molecular, morphology, phylogeny, rRNA, taxonomy

## Abstract

**Simple Summary:**

*Paratylenchus* spp. are vermiform organisms distributed throughout the world that can parasitize a wide variety of cultivated and wild plants. Some species are considered pathogenic in crops; therefore, correct identification is essential to design management strategies. However, the conserved morphology, similarity of morphometric characters and other factors as co-occurrence of more than one species of *Paratylenchus* in the same soil sample hinder identification to species level. In consequence, this identification should be carried out jointly with morphological, morphometrical and molecular data. The present research aims to provide morphological and molecular characterization of some *Paratylenchus* species found in Spain and the description of several new species.

**Abstract:**

In previous studies, fifteen species of *Paratylenchus*, commonly known as pin nematodes, have been reported in Spain. These plant-parasitic nematodes are ectoparasites with a wide host range and global distribution. In this research, 27 populations from twelve *Paratylenchus* species from 18 municipalities in Spain were studied using morphological, morphometrical and molecular data. This integrative taxonomic approach allowed the identification of twelve species, four of them were considered new undescribed species and eight were already known described. The new species described here are *P. caravaquenus* sp. nov., *P. indalus* sp. nov., *P. pedrami* sp. nov. and *P. zurgenerus* sp. nov. As for the already known described species, five were considered as first reports for the country, specifically *P.* *enigmaticus*, *P. hamatus*, *P. holdemani*, *P. israelensis*, and *P. veruculatus*, while *P. baldaccii*, *P. goodeyi* and *P. tenuicaudatus* had already been recorded in Spain. This study provides detail morphological and molecular data, including the D2-D3 expansion segments of 28S rRNA, ITS rRNA, and partial mitochondrial COI regions for the identification of different *Paratylenchus* species found in Spain. These results confirm the extraordinary cryptic diversity in Spain and with examples of morphostatic speciation within the genus *Paratylenchus*.

## 1. Introduction

Pin nematodes of the genus *Paratylenchus* Micoletzky, 1922 [1] are one of the smallest plant-parasitic nematodes; their body length varies from 160 to 600 μm [2]. Stylet length is the organ which drives the feeding habit and morphology of adult females. Some species have a long stylet (>40 μm), become swollen and feeding from deeper layers in the root cortex as sedentary ectoparasites. However, the majority of them feed as migratory ectoparasites on epidermal cells and root hairs [2]. In general, *Paratylenchus* spp. are parasites of higher plants (herbaceous and woody) with a higher abundance in the rhizosphere of trees and perennials [3]. This is probably due to their lifecycle, species with smaller body size and higher fecundity allow for faster build-up of their populations compared to other plant-parasitic species [4,5,6]. Additionally, they can survive using dehydration and be easily dispersed by wind [7]. Many species arrest their development in the fourth-stage juvenile (J4) and some species may molt to adult with root diffusates from their host plant; although some molting might occur in spring in the absence of root exudates [8]. *Paratylenchus* species are widely dispersed in different environments and crops, and world-wide distributed. Pathogenicity has been found only on different crops for a few species such as *P. bukowinensis* Micoletzky, 1922 in celery (*Apium graveolens* L.) [8,9]; *Paratylenchus dianthus* Jenkins & Taylor, 1956 in carnation (*Dianthus caryophyllus* L.) [10]; *Paratylenchus epacris* (Allen & Jensen, 1950) Goodey, 1963 in black walnut (*Juglans nigra* L.) [11]; *Paratylenchus hamatus* Thorne & Allen, 1950 in fig (*Ficus carica* L.), pear (*Pyrus communis* L.) and grapevine (*Vitis vinifera* L.) [12,13,14]; *Paratylenchus microdorus* Andrassy, 1959 in red clover (*Trifolium pratense* L.) and lettuce (*Lactuca sativa* L.) [15]; *Paratylenchus nanus* Cobb, 1923 in garden balsam (*Impatiens balsamina* L.) [16]; *Paratylenchus neoamblycephalus* Geraert, 1965 in Myrobalan plum (*Prunus cerasifera* Ehrh.) [17]; *Paratylenchus projectus* Jenkins, 1956 in alfalfa (*Medicago sativa* L.) and sunflower (*Helianthus annuus* L.) [18,19]; *Paratylenchus shenzhenensis* Wang, Xie, Li, Xu, Yu & Wang, 2013 in *Anthurium andraeanum* Linden ex André [20]; and *Paratylenchus enigmaticus* Munawar, Yevtushenko, Palomares-Rius & Castillo, 2021 in lettuce [21,22]. Other species can be also pathogenic in crops, but further studies are necessary to confirm this. This lack of knowledge is aggravated by the difficulty of working with these tiny nematodes [3]. In this sense, the correct taxonomic identification for putative species damaging crops is of vital importance for their practical management in field. *Paratylenchus* identification to species level is hampered by the largely conserved morphology, overlapping morphometrics, high levels of intra-specific variability and, most importantly, the frequent co-occurrence of more than one pin species in the same soil sample [23,24]. An additional difficulty is that many of these species are found in the soil as quiescent juvenile stages (usually, the fourth-stage juvenile). They could remain in this stage until suitable environmental conditions and/or appropriate plant host are available. Furthermore, Fisher [25] found that morphological characters used for species identification might be influenced by environmental and other factors (such as temperature, host, population size, etc.). For these reasons, it is essential to identify *Paratylenchus* species accurately using integrative taxonomic methods (combination of morphological, morphometrical and molecular data), at least for type populations. Several articles have studied in this genus using this methodology, giving interesting results for their molecular variability and the presence of cryptic species using several populations of the same species [21,23,24,26,27,28,29]. After the characterization of type population of each species, barcoding techniques could be used more easily and effectively in the future for the management of these nematodes in field. Recently, several studies, some using molecular data, have questioned the monophyly of Tylenchulidae [2,30,31,32] and include the genus *Gracilacus* within *Paratylenchus* [2,33,34,35,36]. We follow this last inclusion of all species of *Gracilacus* in *Paratylenchus* as also is showed in recently resolved phylogenies in which the genus *Gracilacus* was distributed along *Paratylenchus* clades [24,28,37]. Likewise, the monotypic genus *Cacopaurus* characterized by the obese female body, tubercles on annuli of the female cuticle and sessile parasitism [38] was synonymized by Goodey [39], but it has been accepted by several authors [2,24,40,41] and in the last monograph of the Tylenchulidae by Ghaderi et al. [2].

Fifteen species of *Paratylenchus* have been reported in Spain from cultivated and wild ecosystems including *P. arculatus* Luc & de Guiran, 1962 [42,43], *P. baldaccii* Raski, 1975 [44], *P. ciccaronei* Raski, 1975 [45,46,47], *P. goodeyi* Oostenbrink, 1953 [48], *P. macrodorus* Brzeski, 1963 [46], *P. microdorus* [44,45,46,47], *P. nanus* Cobb, 1923 [46,49], *P. peraticus* (Raski, 1962) Siddiqi & Goodey, 1964 [48], *P. sheri* (Raski, 1973) Siddiqi, 1986 [45,46,47,49], *P. similis* Khan, Prasad & Mathur, 1967 [46,49], *P. steineri* Golden, 1961 [46,48], *P. straeleni* (De Coninck, 1931) Oostenbrink, 1960 [50], *P. teres* (Raski, 1976) Siddiqi, 1986 [51], *P. tenuicaudatus* Wu, 1961 [52] and *P. vandenbrandei* de Grisse, 1962 [45,47]. However, some studies did not use molecular techniques for their identification and the biodiversity of this group could be underexplored. Therefore, a new assessment of species using molecular barcoding for Spanish populations could be of interests for their unequivocal identification and the reliable estimation of biodiversity. This study tries to understand this biodiversity using an integrative taxonomic approach.

The main objectives of this study were to: (**i**) conduct identification with a morphological and morphometrical approaches of some *Paratylenchus* species collected in several nematode surveys in Spain; (**ii**) provide molecular characterization of several species using ribosomal (D2-D3 expansion segments of 28S rRNA, Internal Transcribed Spacer region (ITS) rRNA) and the mitochondrial region cytochrome c oxidase subunit 1 (COI); and (**iii**) study phylogenetic relationships within *Paratylenchus* spp. using the obtained molecular markers.

## 2. Materials and Methods

### 2.1. Nematode Sampling and Morphological Identification

Soil samples were collected mainly from the rhizosphere of woody plants including pine (*Pinus halepensis* Mill.) and several *Prunus* spp. (almond, apricot, cherry, nectarine and peach) with different rootstocks, in several localities in Spain (Table 1), using a shovel, and considering the upper 5–40 cm depth of soil. Nematodes were extracted from a 500 cm^3^ sub-sample of soil by centrifugal flotation [53].

A total of 231 individuals including 214 females and 17 males were used for morphological and morphometrical analyses. Specimens for study using light microscopy (LM) and morphometrical studies were killed and fixed in an aqueous cold solution of 4% formaldehyde + 1% glycerol, dehydrated using alcohol-saturated chamber and processed to pure glycerine using Seinhorst’s method [54] as modified by De Grisse [55]. Light micrographs were taken using fresh nematodes and measurements of each nematode population including important diagnostic characteristics (i.e., de Man indices, body length, stylet length, lip region, tail shape) [56] were performed using a Leica DM6 compound microscope with a Leica DFC7000 T digital camera using fixed and embedded nematodes in glycerin. Nematodes were identified at the species level using an integrative approach combining molecular and morphological techniques to achieve efficient and accurate identification [21,24,28]. For each nematode population, key diagnostic characters were determined, including body length, stylet length, a ratio (body length/maximum body width), b ratio (body length/total pharynx length), c ratio (body length/tail length), c’ ratio (tail length/body width at anus), V ratio (distance from anterior end to vulva/body length × 100), and o ratio (distance from stylet base to dorsal pharyngeal opening/stylet length 100) [21,24,28], and the sequencing of specific DNA fragments (described below) confirmed the identity of the nematode species for each population.

### 2.2. Nematode Molecular Characterization

For molecular analyses, and in order to avoid mistakes in case of mixed populations in the same sample (being common in several soil samples), single specimens from the sample were temporarily mounted in a drop of 1 M NaCl containing glass beads (to avoid nematode crushing/damaging specimens) to ensure homogenous morphology with specimens conformed with the unidentified population. All necessary morphological and morphometrical data by taking pictures and measurements using the above camera-equipped microscope were recorded. This was followed by DNA extraction from single individuals as described by Palomares-Rius et al. [57], and more importantly, for all the 27 studied isolates, all the three molecular markers of each *Paratylenchus* isolate belong to the same single extracted individual in each PCR tube without any exception. The D2 and D3 expansion domains of the 28S rRNA were amplified using the D2A (5′-ACAAGTACCGTGAGGGAAAGTTG-3′) and D3B (5′-TCGGAAGGAACCAGCTACTA-3′) primers [58]. The Internal Transcribed Spacer region (ITS) was amplified by using forward primer TW81 (5′-GTTTCCGTAGGTGAACCTGC-3′) and reverse primer AB28 (5′-ATATGCTTAAGTTCAGCGGGT-3′) [59]. The COI gene was amplified using the primers JB3 (5′-TTTTTTGGGCATCCTGAGGTTTAT-3′) and JB5 (5′-AGCACCTAAACTTAAAACATAATGAAAATG-3′) [60]. The PCR cycling conditions for the 28S rRNA and ITS regions were as follows: 95 °C for 15 min, followed by 35 cycles of 94 °C for 30 s, an annealing temperature of 55 °C for 45 s, and 72 °C for 1 min, and 1 final cycle of 72 °C for 10 min. The PCR cycling for COI primers was as follows: 95 °C for 15 min, 39 cycles at 94 °C for 30 s, 53 °C for 30 s, and 68 °C for 1 min, followed by a final extension at 72 °C for 7 min. PCR volumes were adapted to 25 μL for each reaction, and primer concentrations were as described in De Ley et al. [58], Subbotin et al. [59] and Bowles et al. [60]. We used 5× HOT FIREpol Blend Master Mix (Solis Biodyne, Tartu, Estonia) in all PCR reactions. The PCR products were purified after amplification using ExoSAP-IT (Affimetrix, USB products, Kandel, Germany) and used for direct sequencing in both directions with the corresponding primers. The resulting products were purified and run in a DNA multicapillary sequencer (Model 3130XL Genetic Analyzer; Applied Biosystems, Foster City, CA, USA), using the BigDye Terminator Sequencing Kit v.3.1 (Applied Bio-systems) at the Stab Vida sequencing facility (Caparica, Portugal). The sequence chromatograms of the 3 markers (ITS, COI and D2-D3 expansion segments of 28S rRNA) were analyzed using DNASTAR LASERGENE SeqMan v. 7.1.0. Basic local alignment search tool (BLAST) at the National Center for Biotechnology Information (NCBI, Bethesda, MD, USA) was used to confirm the species identity of the DNA sequences obtained in this study [61]. The newly obtained sequences were deposited in the GenBank database under accession numbers indicated on the phylogenetic trees and in Table 1.

### 2.3. Phylogenetic Analyses

D2-D3 expansion segments of 28S rRNA, ITS rRNA, and COI mtDNA sequences of the 27 *Paratylenchus* isolates were obtained in this study. These sequences and other sequences from species of *Paratylenchus* from GenBank were used for phylogenetic analyses. Selection of outgroup taxa for each dataset were based on previously published studies [24,28,29]. Multiple sequence alignments of the different genes were completed using the FFT-NS-2 algorithm of MAFFT V.7.450 [62]. BioEdit program V. 7.2.5 [63] was used for sequence alignments visualization and edited by Gblocks ver. 0.91b [64] in Castresana Laboratory server (http://molevol.cmima.csic.es/castresana/Gblocks_server.html (accessed on 27 March 2021)) using options for a less stringent selection (minimum number of sequences for a conserved or a flanking position: 50% of the number of sequences +1; maximum number of contiguous non-conserved positions: 8; minimum length of a block: 5; allowed gap positions: with half). Phylogenetic analyses of the sequence datasets were based on Bayesian inference (BI) using MrBayes 3.1.2 [65]. The best-fit model of DNA evolution was achieved using JModelTest V.2.1.7 [66] with the Akaike Information Criterion (AIC). The best-fit model, the base frequency, the proportion of invariable sites, and the gamma distribution shape parameters and substitution rates in the AIC were then used in MrBayes for the phylogenetic analyses. The general time-reversible model with invariable sites and a gamma-shaped distribution (GTR  + I + G) for the D2-D3 segments of 28S rRNA and the partial ITS rRNA and the general time-reversible model with a gamma-shaped distribution (GTR + G) for COI gene, were run with four chains for 4, 4, and 10 × 10^6^ generations, respectively. A combined analysis of the three ribosomal genes was not undertaken due to several sequences not being available for all species. The sampling for Markov chains was carried out at intervals of 100 generations. For each analysis two runs were conducted. After discarding burn-in samples of 30% and evaluating convergence, the remaining samples were retained for more in-depth analyses. The topologies were used to generate a 50% majority-rule consensus tree. On each appropriate clade posterior probabilities (PP) were given. FigTree software version v.1.42 [67] was used for visualizing trees from all analyses.

## 3. Results

Twelve species were identified from 27 isolates of *Paratylenchus* spp. from 23 soil samples (codified as PR_014, PR_115, PR_118 and PR_193 contain more than one *Paratylenchus* species) in 18 municipalities in Spain. These populations were morphologically studied in detail and molecular markers for their identification were provided (Table 1). From these, 4 were considered new undescribed species and 8 were already known described species (Table 1). The new species described herein include *Paratylenchus caravaquenus* sp. nov., *Paratylenchus indalus* sp. nov., *Paratylenchus pedrami* sp. nov., and *Paratylenchus zurgenerus* sp. nov. The already known species included *Paratylenchus baldaccii*, *Paratylenchus enigmaticus* Munawar, Yevtushenko, Palomares-Rius & Castillo, 2021 [21], *P. goodeyi*, *P. hamatus*, *P. holdemani*, *P. israelensis*, *P. tenuicaudatus*, and *P. veruculatus*. Five of latter are considered as first reports for Spain in this work (*viz*. *P. enigmaticus*, *P. hamatus*, *P. holdemani*, *P. israelensis* and *P. veruculatus*) and measurements and molecular markers are provided for their unequivocal identification.

### 3.1. Systematics

#### 3.1.1. Description of *Paratylenchus caravaquenus* sp. nov.

**Figure 1:**
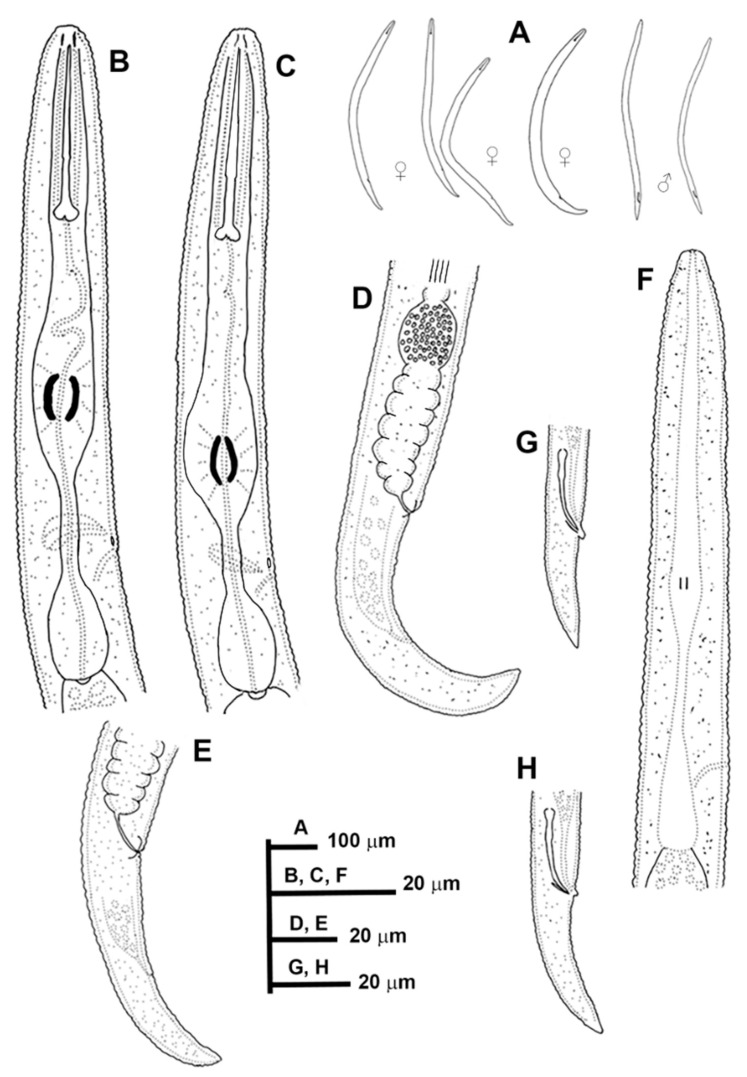
Line drawings of *Paratylenchus caravaquenus* sp. nov. (**A**): Entire females and males; (**B**,**C**): Female pharyngeal region; (**D**,**E**): Female posterior region; (**F**): Male pharyngeal region showing absence of stylet; (**G**,**H**): Male posterior region.

**Figure 2:**
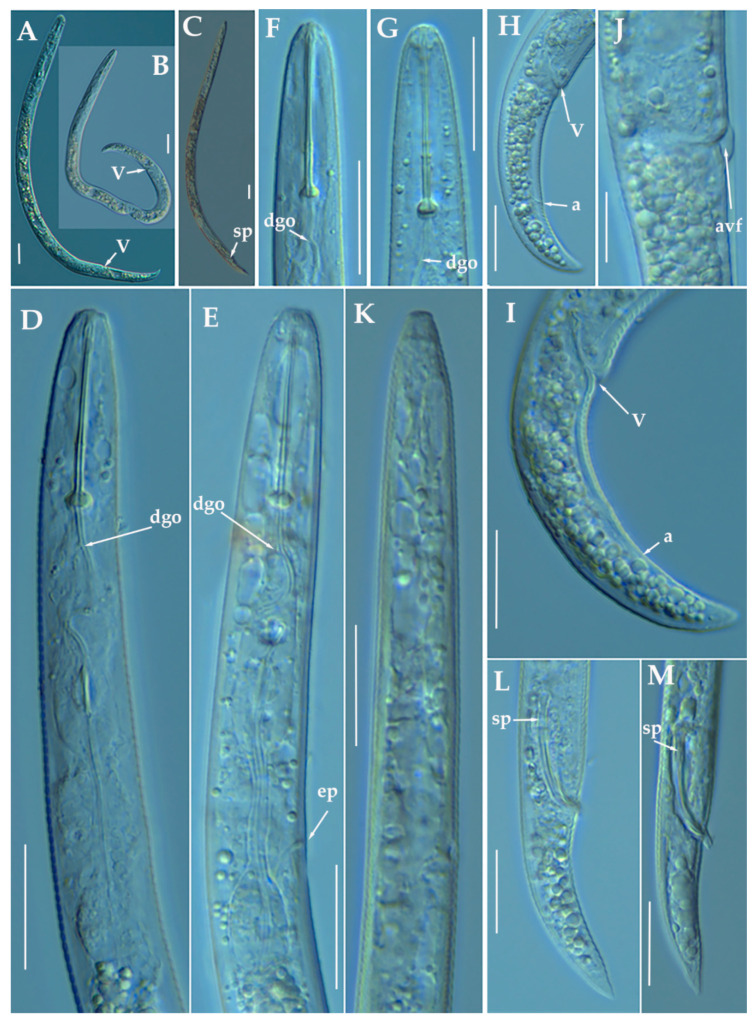
Light photomicrographs of *Paratylenchus caravaquenus* sp. nov. (**A**,**B**): Entire female with vulva arrowed; (**C**): Entire male with spicules arrowed; (**D**,**E**): Female pharyngeal region; (**F**,**G**): Female lip region; (**H**,**I**): Female posterior region with vulva and anus (arrowed); (**J**): Detail of vulva showing advulval flap (arrowed); (**K**): Male pharyngeal region showing absence of stylet; (**L**,**M**): Male posterior region showing spicules (arrowed). Scale bars (**A**–**I**, **K**–**M** = 20 μm; **J** = 10 μm). (Abbreviations: a = anus; avf = advulval flap; dgo = pharyngeal dorsal gland orifice; ep = excretory pore; sp = spicules; V = vulva).

(Figure 1 and Figure 2, Table 2). http://zoobank.org/NomenclaturalActs/50830BAE-BCB4-4465-98D2-27598AB19E61 (accessed on 27 March 2021).

*Female*: Body slender, ventrally arcuate to form an open, C-shaped body habitus when heat relaxed; cuticle finely annulated; lateral field equidistant with four distinct lines; lip region conoid rounded, with anterior end flattened, continuous with the rest of the body, small submedian lobes or almost indistinguishable in some specimens. Labial framework sclerotization weak; pharyngeal region typical paratylenchoid type. Stylet rigid, straight; stylet knobs rounded; dorsal pharyngeal gland opening 5.5–8.0 μm behind stylet knobs. Median pharyngeal bulb slender elongate, bearing distinct large valves; isthmus short slender, surrounded by nerve ring; basal bulb pyriform, pharyngeal-intestinal valve rounded; excretory pore situated at the level or anterior to pharyngeal basal bulb. Hemizonid 1–2 annuli long, situated immediately anterior to excretory pore. Body slightly narrower posterior to vulva; ovary outstretched, well developed; spermatheca and crustaformeria well developed; spermatheca rounded; vulva a transverse slit occupying half of the corresponding body width. Vulval lips prominent, the anterior lip is protruding further than the posterior lip; advulval flaps present, but not prominent in fresh specimens. Anus difficult to distinguish in some specimens; tail slender, conoid, finely annulated, and gradually tapers to form a rounded or subacute terminus in some individuals.

*Male*: Body slender than female, tapering towards both ends, posterior region ventrally arcuate when heat relaxed. Cuticle apparently smooth with fine annulations; labial region similar to that of female but narrower and slightly truncated, continuous with body, sclerotization in labial region weak; stylet lacking. Pharynx rudimentary and non-functional, procorpus, metacorpus, and basal bulb inconspicuous; excretory pore located 73.0 μm away from anterior end. Testis outstretched, with small spermatozoa; spicule slender, slightly curved towards end; gubernaculum curved; bursa absent. Tail elongate-conoid, tapering gradually to a finely pointed tip.

*Juveniles*: It was the most abundant developmental stage at the end of summer in the type locality, most probably J4. They were similar in morphology to the adult females. However, they are characterized by the presence of weak stylet; underdeveloped pharynx components; underdeveloped genital primordium; indistinct anus; and posterior body with a rounded terminus.

##### Diagnosis and Relationships

The new species can be characterized by the presence of 4 lateral lines in lateral field, advulval flaps present, and a moderate female stylet length of 29.8 (26.5–32.0) μm. Lip region conoid-rounded, with the anterior end flattened, continuous with the rest of the body. Excretory pore situated at the level or anterior to the pharyngeal basal bulb. Spermatheca rounded. Tail elongate-conoid gradually tapering to form a rounded terminus. According to species grouping by Ghaderi et al. [36] belongs to group 3 characterized by stylet length less than 40 μm, four lateral lines and advulval flaps present.

Morphologically and morphometrically, the new species is close to *P. baldaccii*, *P. salubris* Raski, 1975, *P. coronatus* Colbran, 1975 and *P. mimulus* Raski, 1975. *Paratylenchus caravaquenus* sp. nov. differs from *P. baldaccii* in having males without stylet vs. males with stylet, other morphometrical characters are in the same range. From a molecular point of view, this species differs from other populations identified as *P. baldaccii* in all the molecular markers studied (D2-D3, ITS and COI). *Paratylenchus caravaquenus* sp. nov. differs from *P. salubris* in tail shape acute to finely rounded vs. usually bluntly rounded, longer female adults (344–443 vs. 200–250 µm), and posterior position of the vulva (V) (81.8–84.5% vs. 78–82%). *Paratylenchus caravaquenus* sp. nov. differs from *P. coronatus* by longer body in females (344–443 vs. 270–300 μm), shorter stylet (26.5–32.0 vs. 31–39 μm) and posterior vulva position (82–84% vs. 78–82%). *Paratylenchus caravaquenus* sp. nov. differs from *P. mimulus* in longer body in females (344–443 μm vs. 180–260 μm) and posterior vulva position (82–84% vs. 78–82%). Other species related phylogenetically with morphological and morphometrical data as *P. holdemani* differs by a shorter body (344–443 μm vs. 290–350 μm), shorter stylet (26.5–32.0 μm vs. 21–23 μm), posterior vulva (81.4–84.5% vs. 84–86%) and males without stylet vs. males with stylet.

##### Molecular Characterization

Three D2-D3 of 28S (MW798270-MW798272), three ITS (MW798316-MW798318), and two COI gene sequences (MW797003-MW797004) were generated for this new species without intraspecific sequence variations. *Paratylenchus caravaquenus* sp. nov. is 97% similar for the D2-D3 region (22 nucleotides and no indels) to *P. nawadus* (MN088373), however, it can be readily morphologically distinguished by longer stylet (26.5–32.0 μm vs. 18.7–22 µm), and the presence of a truncated lip region with well-developed submedian lobes in *P. nawadus* [27]. Unfortunately, no data for ITS or COI from *P. nawadus* are available in the GenBank. From our *P. baldaccii* population (PR_152), *P. caravaquenus* sp. nov. is 79%, 79%, and 84% similar in D2-D3 region, ITS and COI sequences, respectively, clearly separating both species.

##### Remarks

This species has been found in only one forest close to an almond field. The population presented moderate numbers of individuals in soil (224 individuals/500 cm^3^ of soil), but the majority of them were J4 individuals.

##### Type Habitat and Locality

*Paratylenchus caravaquenus* sp. nov. was found in the rhizosphere of a *Pinus halepensis* Mill., 1768 forest (coordinates 38°04′52.6″ N; 2°02′17.0″ W); the municipal district of Caravaca, Murcia, Spain.

##### Etymology

The species epithet, *caravaquenus*, refers to the name of the type locality (Caravaca).

##### Type Material

Holotype female, 17 paratypes females and 6 male paratypes (slide numbers PI_AR-01 to PI_AR-11) were deposited in the Nematode Collection of the Institute for Sustainable Agriculture, CSIC, Córdoba, Spain, and two females deposited at the USDA Nematode Collection (slide T-7479p).

#### 3.1.2. Description of *Paratylenchus indalus* sp. nov.

**Figure 3:**
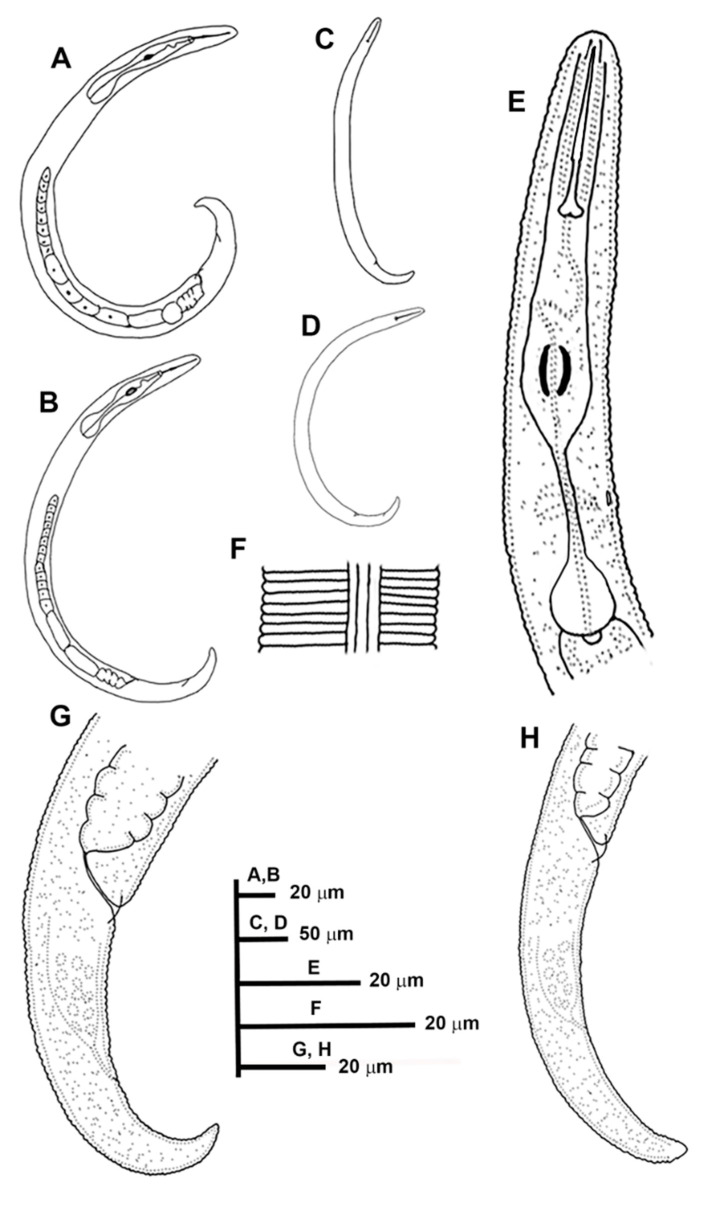
Line drawings of *Paratylenchus indalus* sp. nov. (**A**–**D**): Entire females; (**E**): Female pharyngeal region; (**F**): Lateral field at mid-body; (**G**,**H**): Female posterior region.

**Figure 4:**
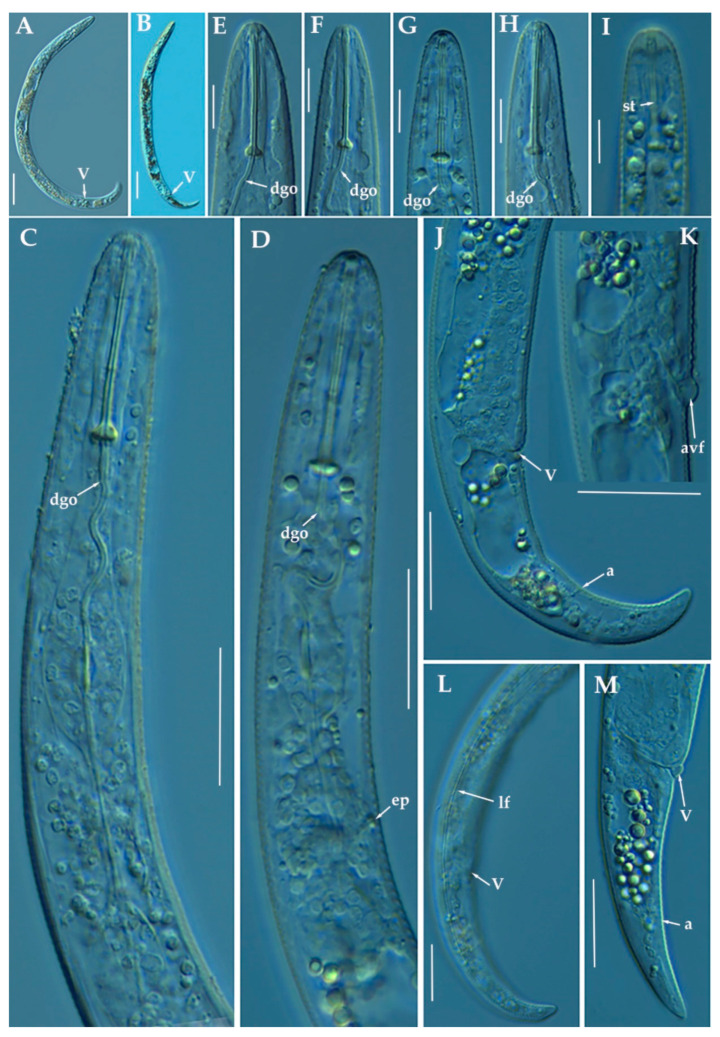
Light photomicrographs of female of *Paratylenchus indalus* sp. nov. (**A**,**B**): Entire female with vulva arrowed; (**C**,**D**): Female pharyngeal region; (**E**–**H**): Female lip region; (**I**): Fourth-stage juvenile showing stylet (arrowed); (**J**): Female posterior region with vulva and anus (arrowed); (**K**): Detail of vulva showing advulval flap (arrowed); (**L**,**M**): Female posterior region with lateral field, vulva and anus (arrowed). Scale bars (**A**–**D**, **J**–**M** = 20 μm; **E**–**I** = 10 μm). (Abbreviations: a = anus; avf= advulval flap; dgo = pharyngeal dorsal gland orifice; ep = excretory pore; lf = lateral field; V = vulva).

**Figure 5:**
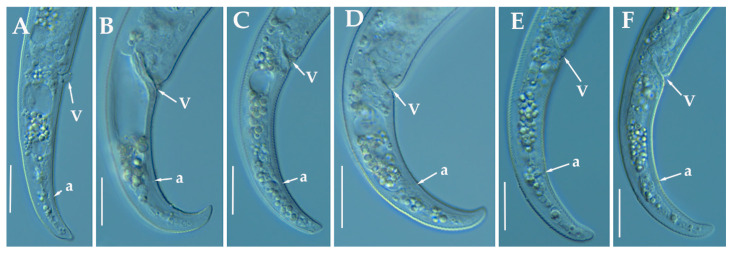
Light photomicrographs of *Paratylenchus indalus* sp. nov. female posterior regions (**A**–**F**). Scale bar: 20 μm). (Abbreviations: a = anus; V = vulva).

(Figure 3, Figure 4 and Figure 5; Table 3). http://zoobank.org/NomenclaturalActs/AD30DC56–76E0-4741-A38A-52734155F641 (accessed on 27 March 2021).

*Female*: Body slender, ventrally arcuate to form an open C-shaped when heat relaxed. Cuticle finely annulated; lateral field equidistant with four distinct lines. Lip region rounded, with anterior end flattened, continuous with the rest of the body, presence of small submedian lobes; labial framework sclerotization weak. Pharyngeal region typical paratylenchoid type; stylet rigid, straight; stylet knobs rounded; dorsal pharyngeal gland opening 5.5–6.5 μm behind stylet knobs. Median pharyngeal bulb slender elongate, bearing distinct large valves; isthmus short slender, surrounded by nerve ring; basal bulb pyriform, pharyngeal-intestinal valve rounded; excretory pore situated anterior to pharyngeal basal bulb. Hemizonid 1–2 annuli long situated immediately anterior to excretory pore; body slightly narrower posterior to vulva; ovary outstretched, well developed. Spermatheca and crustaformeria well developed; spermatheca rounded; vulva a transverse slit occupying half of the corresponding body width; vulval lips prominent, the anterior lip is protruding further than the posterior lip; advulval flaps present, but not prominent in fresh specimens. Anus difficult to distinguish. Tail slender, conoid, finely annulated, and gradually tapers to form a rounded terminus or pointed in some individuals (Figure 5).

*Male*: Not found.

*Juveniles*: It is the most abundant developmental stage at the end of summer in the type locality and other localities. They were similar in morphology to females. However, they are characterized by the presence of a weak stylet; underdeveloped pharynx components; underdeveloped genital primordium; indistinct anus; and posterior body with a rounded or pointed terminus.

##### Diagnosis and Relationships

The new species can be characterized by the presence of 4 lateral lines, advulval flaps, and a stylet length of 28.3 (26.0–29.5) µm. Lip region rounded, with the anterior end flattened, continuous with the rest of the body. Excretory pore situated at the level or anterior to the pharyngeal basal bulb. Spermatheca rounded. Tail conoid gradually tapering to form a rounded terminus.

Morphologically, the new species is close to *P. projectus, P. neoprojectus* Wu & Hawn, 1975 and *P. enigmaticus*. From these species no important and clear morphological and morphometrical differences can be detected. However, *P. projectus* and *P. neoprojectus* have important differences molecularly to *P. indalus* sp. nov. Molecular differences using population paratypes from *P. projectus* and *P. neoprojectus* must be necessary in order to separate these three closely related morphologically species. *Paratylenchus projectus* differs from *P. neoprojectus* by having a trapezoid-shaped lip region, more anterior position of excretory pore and often digitate tail terminus, but these characters could have some variation [36]. These species are also similar to *P. nanus* and *P. neoamblycephalus* from which differ by having empty spermatheca and absence of males [36]. Molecularly, this species is closely related but different to paratypes from *P. enigmaticus*. This is a case of cryptic speciation among these three species comprising a complex group of pin nematodes.

##### Molecular Characterization

Ten D2-D3 of 28S rRNA (MW798273-MW798282), ten ITS (MW798319- MW798328), and four COI gene sequences (MW797005-MW797008) from the four different populations were generated herein for this species. All sequences showed no intraspecific variation, except for the ITS sequences, where one variable position was found. *Paratylenchus indalus* sp. nov. was closely related with *P. enigmaticus*, showing similarity values of 96% (differing by 25 nucleotides and 1 indel) for the D2-D3 region with several accession from *P. enigmaticus*, such as MN535546 or MW282761. For the ITS region, the similarity values were from 94 to 96% (differing by 24 to 39 nucleotides and from 10 to 15 indels) with the *P. enigmaticus* accessions MW282773 and MN535549, respectively. Finally, the similarity found for the COI gene sequences was 97% (differing by 11 nucleotides) with *P. enigmaticus* accessions (MW421686, MN782403 and MW316640). *Paratylenchus indalus* sp. nov. is not so closely molecularly related to *P. projectus* and *P. neoprojectus* as for *P. enigmaticus*, with a similarity of 92–93%, 89% and 87% for D2-D3 of 28S rRNA, ITS region and COI, respectively.

##### Remarks

This species has been found in four almond orchards with undetermined rootstock in four different localities in Almería province (South-eastern Spain). The population presented moderate to high numbers of individuals in soil (from 68 to 2268 individuals/500 cm^3^ of soil), being the majority of them J4 at the end of summer.

##### Type Habitat and Locality

*Paratylenchus indalus* sp. nov. was found in the rhizosphere of almond at Santa María de Nieva, Almería province (coordinates 37°35′26.8″ N; 2°02′03.5″ W), and also has been found in four localities in Almería province (Table 1).

##### Etymology

The species epithet, *indalus*, is derived from the name ‘indalo’ a prehistoric symbol found in a cave of Almería, the province of the locality where the type specimens were collected.

##### Type Material

Holotype female, and 11 paratypes females (slide numbers PR_104–01 to PR_104–06) were deposited in the Nematode Collection of the Institute for Sustainable Agriculture, CSIC, Córdoba, Spain, and two females deposited at the USDA Nematode Collection (slide T-7480p).

#### 3.1.3. Description of *Paratylenchus pedrami* sp. nov.

**Figure 6:**
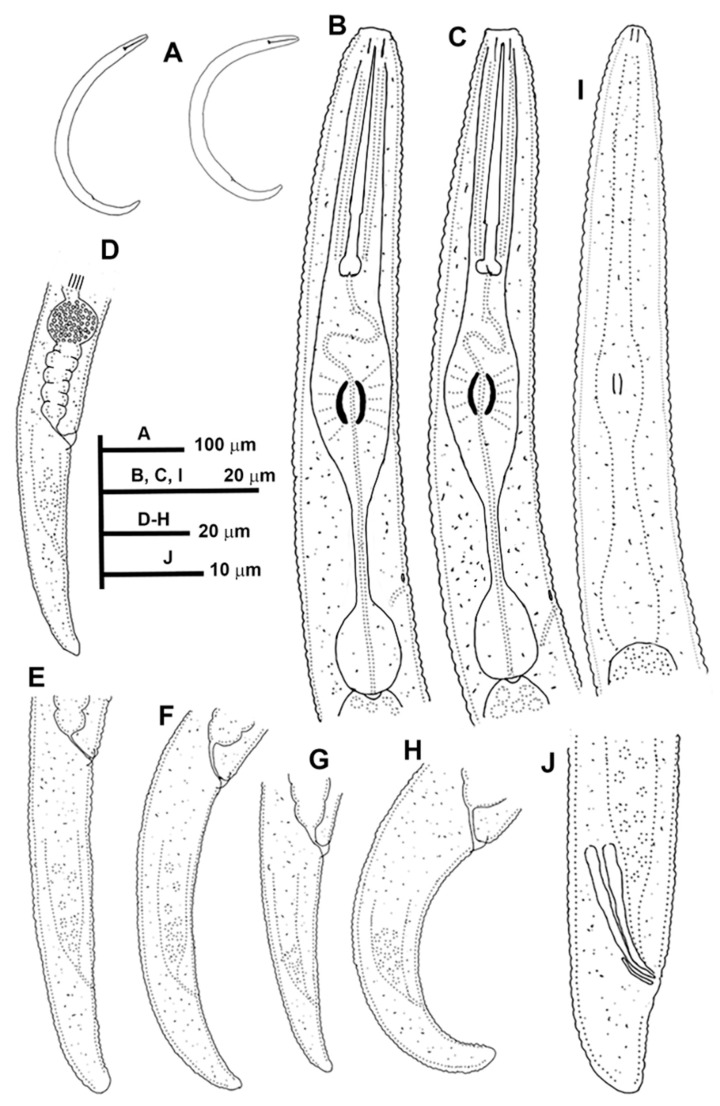
Line drawings of *Paratylenchus pedrami* sp. nov. (**A**): Entire females; (**B**,**C**): Female pharyngeal region; (**D**–**H**): Female posterior region; (**I**): Male pharyngeal region showing absence of stylet; (**J**): Male posterior region.

**Figure 7:**
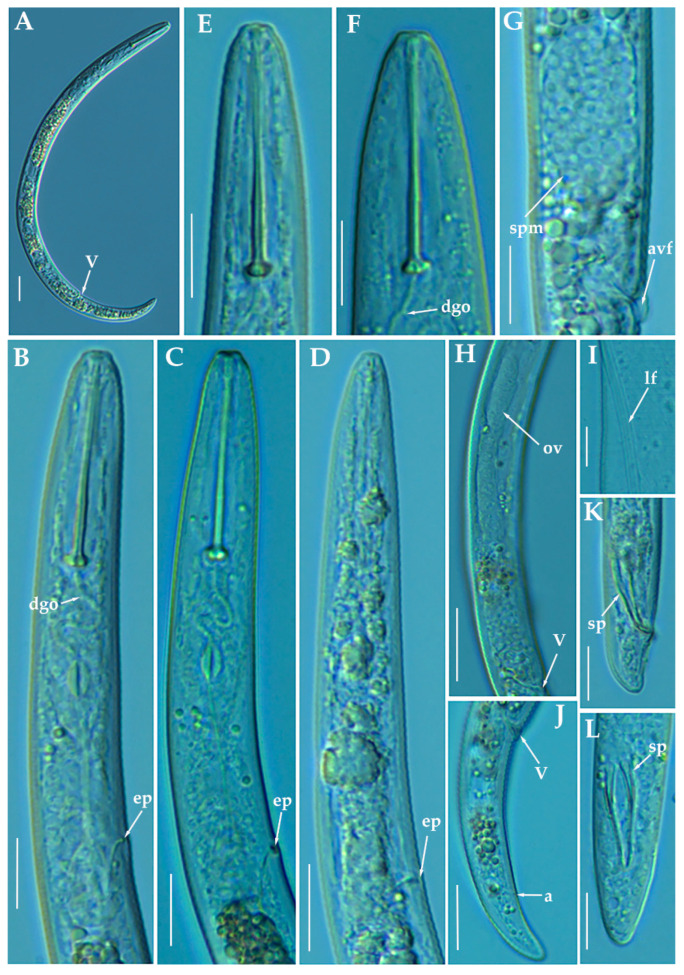
Light photomicrographs of female of *Paratylenchus pedrami* sp. nov. (**A**): Entire female with vulva arrowed; (**B**,**C**): Female pharyngeal region; (**D**): Male pharyngeal region; (**E**,**F**): Female lip region; (**G**): Vulval region showing spermatheca (arrowed); (**H**): Female posterior region showing complete genital branch; (**I**): Detail of lateral field at mid-body (arrowed); (**J**): Female posterior region with vulva and anus arrowed; K, L: Male posterior region with spicules (arrowed). Scale bars (**A**,**H**,**J** = 20 μm; **B**–**G**,**I**,**K**,**L** = 10 μm). (Abbreviations: a = anus; avf = advulval flap; dgo = pharyngeal dorsal gland orifice; ep = excretory pore; lf = lateral field; ov = ovary; spm = spermatheca; V = vulva).

**Figure 8:**
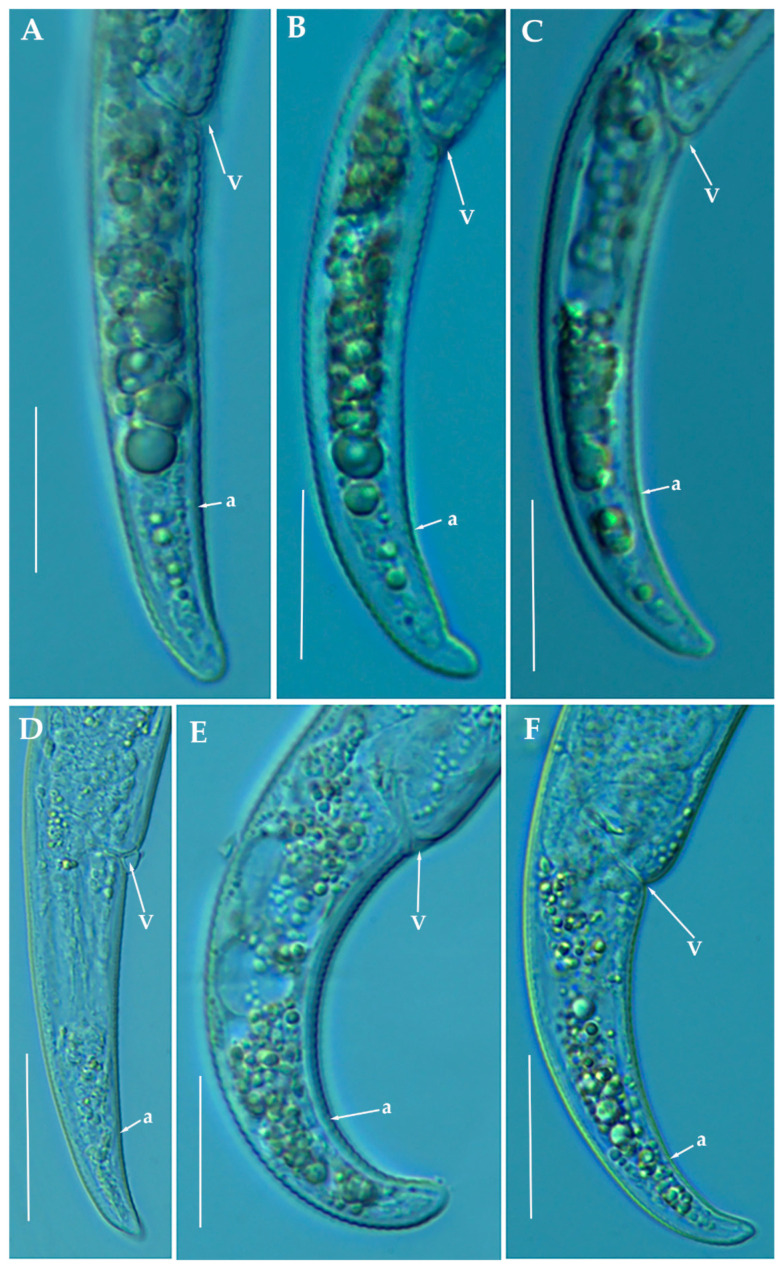
Light photomicrographs of *Paratylenchus pedrami* sp. nov. female posterior regions (**A**–**F**). Scale bars (**A**–**F** = 20 μm). (Abbreviations: a = anus; V = vulva).

(Figure 6, Figure 7 and Figure 8; Table 4). http://zoobank.org/NomenclaturalActs/1ABA6B7F-79FB-4AAF-BD9D-8765183A3353 (accessed on 27 March 2021).

*Female*: Body slender, ventrally arcuate to form an open C-shaped when heat relaxed. Cuticle finely annulated; lateral field equidistant with four distinct lines. Lip region rounded, with anterior end flattened, continuous with the rest of the body, presence of small submedian lobes. Labial framework sclerotization weak; pharyngeal region typical paratylenchoid type. Stylet rigid, straight; stylet knobs rounded; dorsal pharyngeal gland opening 3.5–5.0 μm behind stylet knobs. Median pharyngeal bulb slender elongate, bearing distinct large valves; isthmus short slender, surrounded by nerve ring; basal bulb pyriform, pharyngeal-intestinal valve rounded; excretory pore situated at the level or anterior to pharyngeal basal bulb. Hemizonid 1–2 annuli long situated immediately anterior to excretory pore; body slightly narrower posterior to vulva; ovary outstretched, well developed. Spermatheca and crustaformeria well developed; spermatheca rounded; vulva a transverse slit occupying half of the corresponding body width; advulval lips prominent, the anterior lip is protruding further than the posterior lip; vulval flaps present, but not prominent in fixed specimens. Anus difficult to distinguish. Tail slender, conoid, finely annulated, and gradually tapering to form a rounded terminus or pointed in some individuals (Figure 8).

*Male*: Body slender than female, tapering towards both ends, cuticle apparently smooth with fine annulations; labial region similar to that of female but narrower and slightly truncated, continuous with body, sclerotization in labial region weak. Stylet lacking; pharynx rudimentary, procorpus, metacorpus, and basal bulb inconspicuous, and non-functional. Excretory pore located 65.5 μm from anterior end. Testis outstretched, with small spermatozoa; spicule slender, slightly curved towards end; gubernaculum curved; bursa absent; tail short and rounded.

*Juveniles*: It is the most abundant developmental stage at the end of summer in the type locality and other localities. They were similar in morphology to adult females. However, they are characterized by the presence of weak stylet; underdeveloped pharynx components; underdeveloped genital primordium; indistinct anus; and posterior body with a rounded or pointed terminus.

#### Diagnosis and Relationships

The new species can be characterized by the presence of 4 lateral lines, advulval flaps, and a stylet length of 28.3 (26.0–29.5) µm. Lip region rounded, with the anterior end flattened, continuous with the rest of the body. Excretory pore situated at the level or anterior to the pharyngeal basal bulb. Spermatheca rounded. Tail conoid gradually tapering to form a rounded terminus. Males with a short and rounded tail. According to species grouping by Ghaderi et al. [36] belongs to group 3 characterized by stylet length less than 40 µm, four lateral lines and advulval flaps present.

Morphologically, the new species is close morphologically and morphometrically to *P. baldaccii*, *P. salubris*, *P. coronatus* and *P. mimulus*. *Paratylenchus pedrami* sp. nov. differs from *P. baldaccii* in shape of the male tail, short and rounded vs. conical and pointed, and absence vs. presence of male stylet, slightly shorter body of females (231–374 µm vs. 280–430 µm) and different molecular markers studied in this article. A single male specimen of *P. pedrami* sp. nov. was sequenced for confirming these morphological differences with *P. baldaccii. Paratylenchus pedrami* sp. nov. differs from *P. salubris* in shorter female stylet (26.0–30.0 vs. 28.0–35.0 µm) and male tail shape, short and rounded vs. conical and pointed. *Paratylenchus pedrami* sp. nov. differs from *P. coronatus* in shorter female stylet (26.0–30.0 µm vs.31–39 µm), in male tail shape (rounded vs. conoid-arcuate), and different molecular markers deposited in GenBank. *Paratylenchus pedrami* sp. nov. differs from *P. mimulus* in longer body of females (231–374 µm vs. 180–260 µm) and different male tail shape (rounded vs. conoid with finely rounded terminus).

#### Molecular Characterization

Two populations from this new species were molecularly characterized, including three identical D2-D3 of 28S rRNA (MW798283-MW798285), two identical ITS sequences (MW798329-MW798330) and one COI gene sequences (MW797009). The closest *Paratylenchus* sequences were from *P. baldaccii* with 96%, 93% and 90% similarity (differing by 30, 45 and 26 nucleotides) for the D2-D3 of 28S, ITS region, and COI gene, respectively.

#### Remarks

This species has been found in two almond orchards with undetermined rootstock in two different localities in Córdoba province (Southern Spain). The population presented moderate numbers of individuals in soil (200 and 216 individuals/500 cm^3^ of soil), being the majority of them J4 at the end of summer.

*Paratylenchus pedrami* sp. nov. was found in the rhizosphere of almond at Córdoba, Córdoba province (coordinates 37°49′39.9″ N; 4°53′22.0″ W), and also has been found in other place at the same locality (Table 1), both separated by approximately two kilometers.

#### Etymology

The species epithet, *pedrami*, is dedicated to Dr. Majid Pedram, an important Iranian nematologist from Department of Plant Pathology, Faculty of Agriculture, Tarbiat Modares University, Tehran, Iran.

#### Type Material

Holotype female, and 18 paratypes females (slide numbers PR_014–01 to PR_014–09) were deposited in the Nematode Collection of the Institute for Sustainable Agriculture, CSIC, Córdoba, Spain, and two females deposited at the USDA Nematode Collection (slide T-7481p).

### 3.1.4. Description of *Paratylenchus zurgenerus* sp. nov.

**Figure 9:**
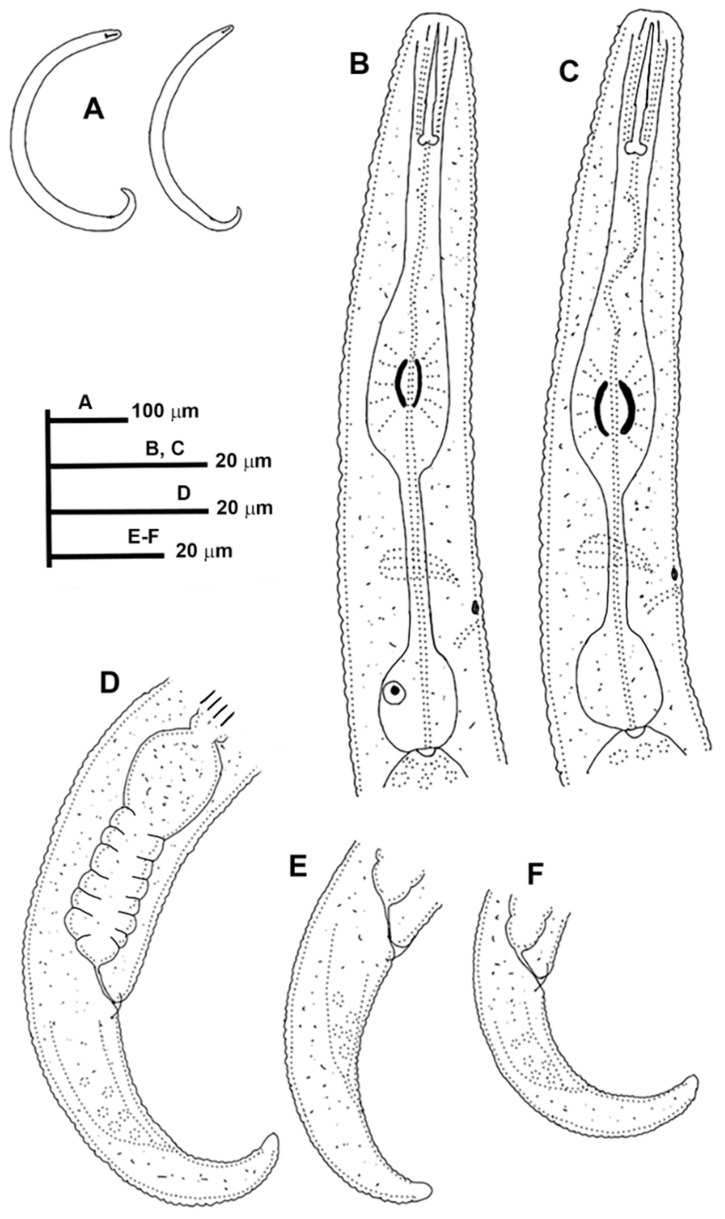
Line drawings of *Paratylenchus zurgenerus* sp. nov. (**A**): Entire females; (**B**,**C**): Female pharyngeal region; (**D**–**F**): Female posterior region.

**Figure 10:**
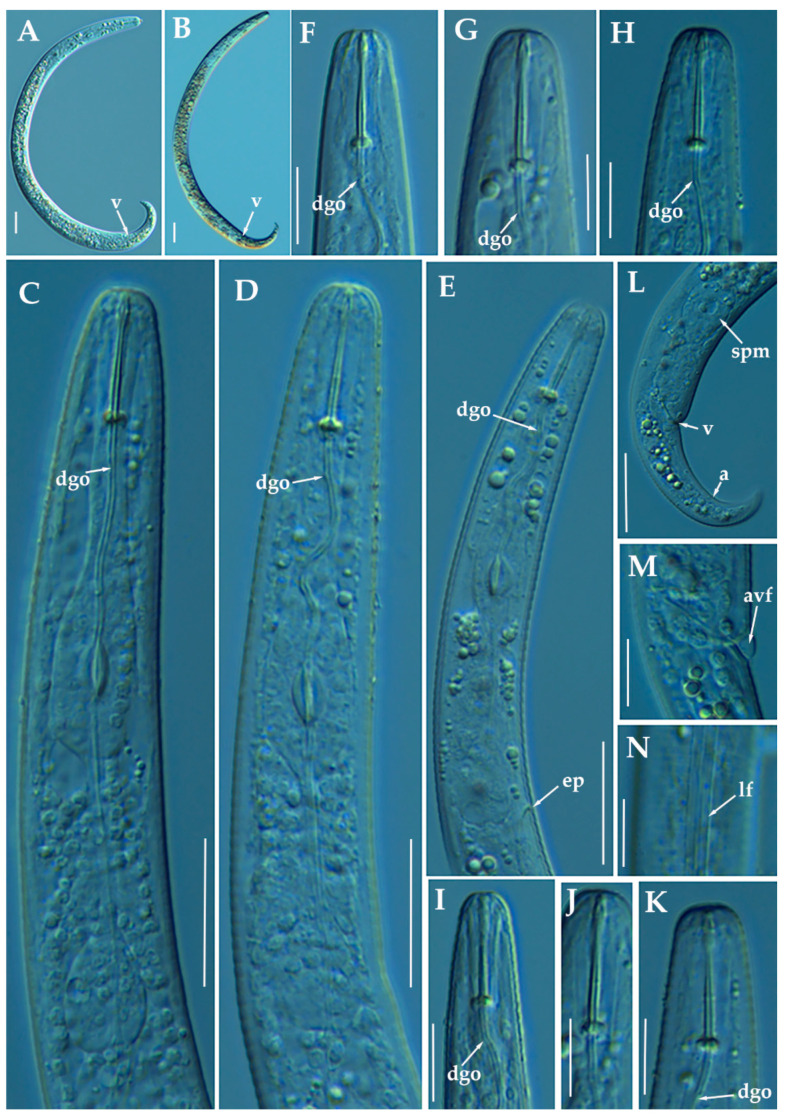
Light photomicrographs of female of *Paratylenchus zurgenerus* sp. nov. (**A**,**B**): Entire female with vulva arrowed; (**C**–**E**): Female pharyngeal region; (**F**–**K**): Female lip region; (**L**): Female posterior region showing empty spermatheca, vulva and anus (arrowed); (**M**): Detail of vulva showing advulval flap (arrowed); (**N**): Female mid-region with lateral field (arrowed). Scale bars (**A**–**E**,**L** = 20 μm; **F**–**K**,**M**,**N** = 10 μm). (Abbreviations: a = anus; avf = advulval flap; dgo = pharyngeal dorsal gland orifice; ep = excretory pore; lf = lateral field; spm = spermatheca; V = vulva).

**Figure 11:**
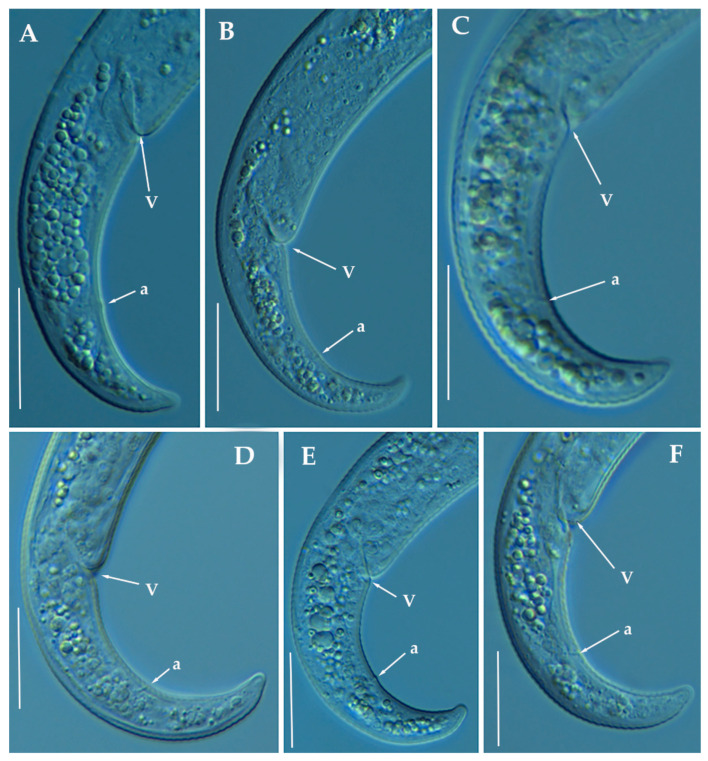
Light photomicrographs of *Paratylenchus zurgenerus* sp. nov. female posterior regions (**A**–**F**). Scale bars (**A**–**F** = 20 μm). (Abbreviations: a = anus; V = vulva).

(Figure 9, Figure 10 and Figure 11; Table 5). http://zoobank.org/NomenclaturalActs/6BE06C25-B8CB-4057-B2DE-3118A314073D (accessed on 27 March 2021).

*Female*: Body slender, ventrally arcuate to form a C-shaped when heat relaxed. Cuticle finely annulated; lateral field equidistant with four distinct lines. Lip region rounded, with anterior end flattened, continuous with the rest of the body, absence of submedian lobes. Labial framework sclerotization relatively strong; pharyngeal region typical paratylenchoid type. Stylet delicate, straight; stylet knobs rounded; dorsal pharyngeal gland opening 3.5–5.0 μm behind stylet knobs. Median pharyngeal bulb slender elongate, bearing distinct large valves; isthmus short slender, surrounded by nerve ring; basal bulb pyriform, pharyngeal-intestinal valve rounded. Excretory pore situated at the level or anterior to pharyngeal basal bulb; hemizonid 1–2 annuli long situated immediately anterior to excretory pore; body slightly narrower posterior to vulva. Ovary outstretched, well developed; spermatheca and crustaformeria well developed; spermatheca rounded; vulva a transverse slit occupying half of the corresponding body width; vulval lips prominent, the anterior lip is protruding further than the posterior lip; vulval flaps present, but not prominent in fresh specimens. Anus difficult to distinguish (more distinguishable in alive specimens). Tail slender, conoid, finely annulated, and gradually tapering to form a rounded terminus or pointed in some individuals (Figure 11).

*Male*: Not found.

*Juveniles*: It is the most abundant developmental stage at the end of summer in the type locality. Fourth-life stage of individuals was similar in morphology to the adult females. However, they are characterized by the presence of a weak stylet (10–11 µm); underdeveloped pharynx components; underdeveloped genital primordium; indistinct anus; and posterior body with a rounded or pointed terminus.

#### Diagnosis and Relationships

The new species can be characterized by the presence of 4 lateral lines, advulval flaps, and a short and delicate stylet length of 15.4 (14.0–16.0) µm. Lip region rounded, with the anterior end flattened, continuous with the rest of the body. Excretory pore situated at the level or anterior to the pharyngeal basal bulb. Spermatheca rounded. Tail conoid gradually tapering to form a rounded terminus. According to species grouping by Ghaderi et al. [36] belongs to group 3 characterized by stylet length less than 40 µm, four lateral lines and advulval flaps present.

Morphologically, the new species is close to *P. microdorus*, *Paratylenchus recisus* Siddiqi, 1996, and *P. veruculatus*. *Paratylenchus zurgenerus* sp. nov. differs from the original type population of *P. microdorus* in a posterior position of the vulva (83.3–86.6% vs. 81–82%), smaller c’ ratio (2.0–3.2 vs. 4.5), and posterior position of excretory pore (67.0–94.0 µm vs. 65 µm). Tail in *P. microdorus* is variable, pointed or rounded, and specimens with various tail ends have been found in almost every population examined [36], but our populations showed a prominent rounded tip. Molecular data of *P. microdorus* [24] clearly differs from our population of *P. zurgenerus* sp. nov. (see below). *Paratylenchus zurgenerus* sp. nov. differs from the original type population of *P. recisus* in longer body (316–418 µm vs. 270–390 µm), posterior position of the vulva (83.3–86.6% vs. 78–83%), and wider lip region (6.0–8.0 µm vs. 5.0–5.2 µm).

#### Molecular Characterization

Four D2-D3 of 28S rRNA (MW798286-MW798289), and four ITS sequences (MW798331-MW798334) were obtained for this species. In both ribosomal genes, no intraspecific variability was detected between three of the four individuals sequenced, whereas in the fourth one, 11 different nucleotides were detected for the D2-D3 (MW798289) and 24 nucleotides and 4 indels for the ITS region (MW798334). Two identical COI gene sequences (MW797010-MW797011) were generated for *P. zurgenerus* sp. nov. The D2-D3 sequences were found to be 99% similar (differing from 1 to 11 nucleotides) and in the same phylogenetic clade of *Paratylenchus* sp.7 (KF242242) from California (USA) that should be consider as conspecific to *P. zurgenerus* sp. nov., however, only molecular and no morphological data are available for this species [23]. The closest *Paratylenchus* spp. for the rest of the molecular markers studied was *P. microdorus* (MW413599-MW413600) being 87% similar for the ITS region (differing by 38 to 41 nucleotides and from 28 to 31 indels) and 91% for COI sequences (MW421666-MW421667) (differing by 28 nucleotides and no indels).

#### Remarks

This species has been found in one almond orchard with undetermined rootstock in one locality in Granada province (Southern Spain). The population presented moderate-high numbers of individuals in soil (1470 individuals/500 cm^3^ of soil), being the majority of them J4 at the end of summer. Probably this species could be the same previously described by Gomez-Barcina et al. [45] as *P. microdorus* as its measurements are identical and in geographically related areas in Southern Spain, but further studies are needed for confirming this hypothesis.

#### Type Habitat and Locality

*Paratylenchus zurgenerus* sp. nov. was only found in the rhizosphere of almond at Zurgena, Almería province (coordinates 37°24′22.5″ N 2°02′00.3″ W) (Table 1).

#### Etymology

The species epithet, *zurgenerus*, refers to the name of the gentilice of inhabitants of the type locality (Zurgena).

#### Type Material

Holotype female, and 17 paratypes females (slide numbers PR_111–01 to PR_111–09) were deposited in the Nematode Collection of the Institute for Sustainable Agriculture, CSIC, Córdoba, Spain, and two females deposited at the USDA Nematode Collection (slide T-7482p).

#### 3.1.5. Morphometrics and Remarks of Known *Paratylenchus* Spanish Populations

Morphometrical data as well as molecular characterization of other already known *Paratylenchus* populations detected in the present study were compared with original and previous reported populations, and these species included *P. baldaccii*, *P. enigmaticus*, *P. goodeyi*, *P. hamatus*, *P. holdemani*, *P. israelensis*, *P. tenuicaudatus*, and *P. veruculatus*.

*Paratylenchus enigmaticus* Munawar, Yevtushenko, Palomares-Rius & Castillo, 2021.

**Figure 12:**
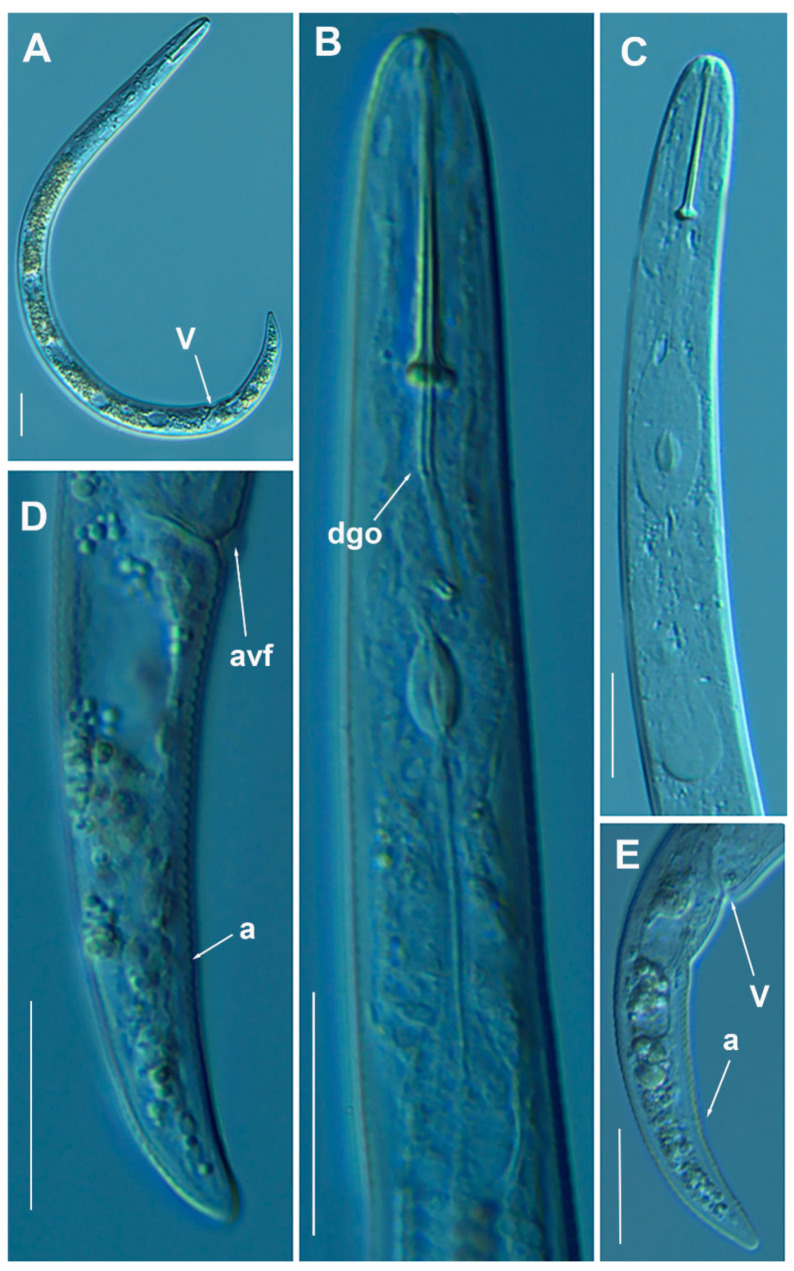
Light photomicrographs of *Paratylenchus enigmaticus* Munawar, Yevtushenko, Palomares-Rius & Castillo, 2021. (**A**): Entire female with vulva arrowed; (**B**,**C**): Female pharyngeal region; (**D**,**E**): Female posterior region showing vulva and anus (arrowed). Scale bars (**A**–**E** = 20 μm). (Abbreviations: a = anus; avf = advulval flap; dgo = pharyngeal dorsal gland orifice; V= vulva).

This species has been recently described from Canada [21]; and it has also been reported in Belgium [22,24]. In the present study, the *P. enigmaticus* population from Spain matches with the original species description, except for minor differences in body length and a ratio; the Spanish population is slightly shorter than the original one (324–383 µm vs. 343–431 µm) and with smaller a ratio (17.6–21.6 vs. 21.7–28.7) (Table 6, Figure 12). This population is closely related morphometrically to the T1-T5 populations found in Belgium [22]. This species has not been reported before in Spain and constitutes the first record for the country.

*Molecular characterization*: Molecular markers agree with the identification of this species. One D2-D3 of 28S rRNA (MW798292), one ITS (MW798337) and one COI gene sequences (MW797013) were generated for this species, and all of them were found to be identical to several accessions from *P. enigmaticus* deposited in GenBank such as, MN535546 for D2-D3 of 28S, MW319816 and MN535551 for the ITS region, and MW421686 for the COI gene sequences.

*Paratylenchus goodeyi* (Oostenbrink, 1953) Raski, 1962.

This species has been detected in several countries as the Netherlands [68,69], Belgium, Germany, and England [24,69,70], Kazakhstan [71], Moldavia [72], Karelia (Russia) [73], Spain [48], Poland [33,74,75], and Slovakia [76]. However, only measurements are presented in Oostenbrink [68], Szczygiel [74], Castillo et al. [48], Brzeski [33,75] and Singh et al. [24].

**Figure 13:**
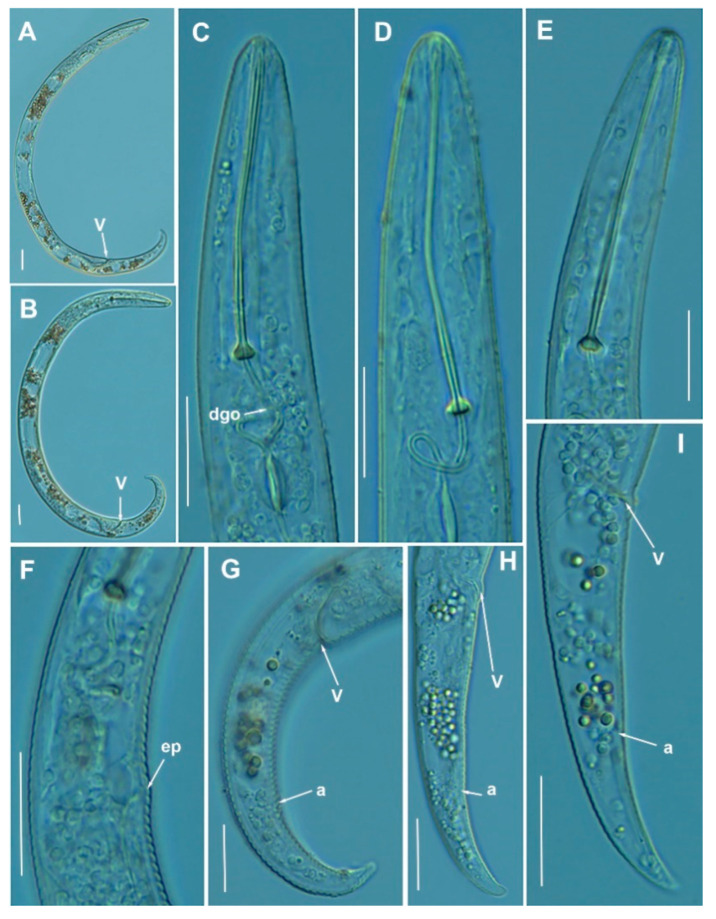
Light photomicrographs of *Paratylenchus goodeyi* (Oostenbrink, 1953) Raski, 1962. (**A**,**B**): Entire female with vulva arrowed; (**C**–**E**): Female lip region; (**F**): Detail of excretory pore; (**G**–**I**): Female posterior region with vulva and anus (arrowed). Scale bars (**A**–**I** = 20 μm). (Abbreviations: a = anus; dgo = pharyngeal dorsal gland orifice; ep = excretory pore; V = vulva).

The Spanish population from Córdoba characterized in this study coincides mainly with the original description of the species (Table 6, Figure 13) at exception of the position of the excretory pore (82.0–102.0 µm vs. 64 µm). This species matches well with other populations, as the Spanish population described by Castillo et al. [48], at exception of a shorter body [396–427 µm vs. 410–450 µm] and smaller c’ ratio (3.2–3.8 vs. 4.1–4.7).

*Molecular characterization*: Molecular markers of this population agree with those provided for this species by Singh et al. [24]. Two D2-D3 of 28S rRNA (MW798293-MW798294), two ITS (MW798338- MW798339) and two COI gene sequences (MW797014-MW797015) were generated in this study without intraspecific sequence variations for this population. The D2-D3 of 28S rRNA sequences were 99% similar (differing by 9 nucleotides) with *P. goodeyi* from Belgium (MW413631-MW413633). The ITS sequences were 96% similar (differing by 27 nucleotides and 11 indels) with the *P. goodeyi* sequence MW423594 and finally, the COI gene sequences showed 95% similarity (differing by 19 nucleotides) with the accessions from *P. goodeyi* deposited in GenBank (MW421648-MW421649).

*Paratylenchus hamatus* Thorne & Allen, 1950 and *Paratylenchus baldaccii* Raski, 1975.

**Figure 14:**
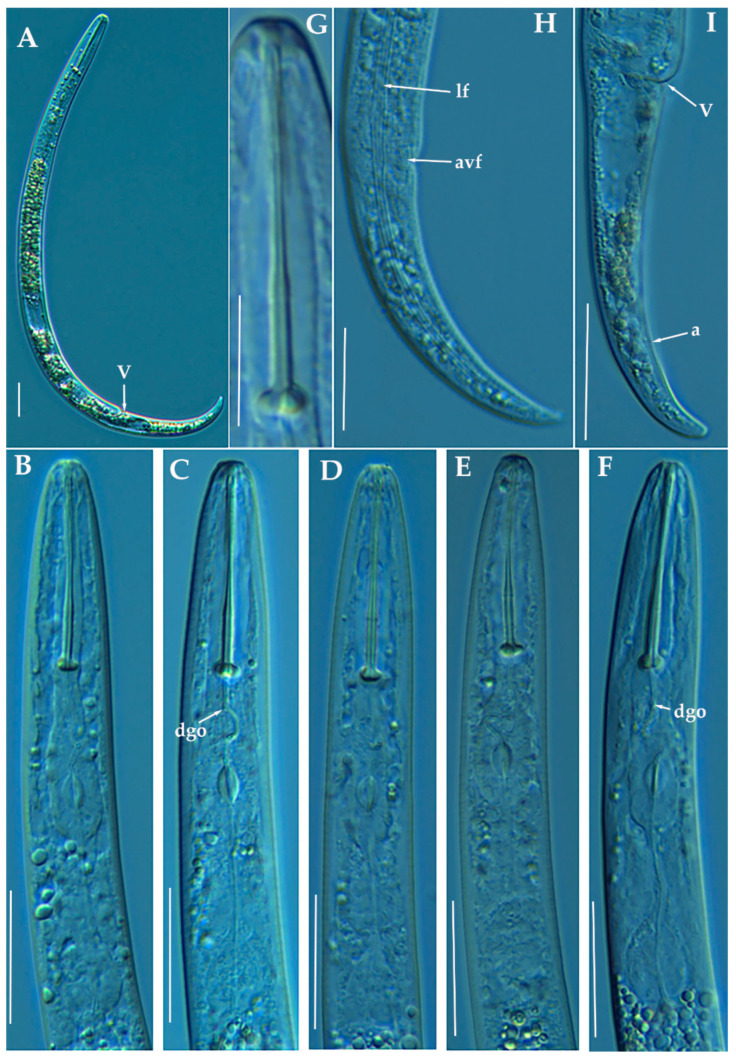
Light photomicrographs of *Paratylenchus baldaccii* Raski, 1975. (**A**): Entire female with vulva arrowed; (**B**–**F**): Female pharyngeal region; (**G**): Detail of female stylet; (**H**): Female posterior region showing lateral field and advulval flap (arrowed); (**I**): Female posterior region showing vulva and anus (arrowed). Scale bars (**A**–**F**, **H**,**I** = 20 μm; **G** = 10 μm). (Abbreviations: a = anus; avf = advulval flap; dgo = pharyngeal dorsal gland orifice; lf = lateral field; V = vulva).

These species are closely related morphologically to other species such as *P. tenuicaudatus.* Van den Berg et al. [23] included *P. tenuicaudatus* within the *P. hamatus sensu stricto*, *P. baldaccii* and two other putative species within the *P. hamatus* “species complex”. Only one character has been pointed by different authors to separate *P. hamatus* from *P. baldaccii*, *viz*. slenderer and sharply conoid female tail tip and male tail tips in *P. hamatus* vs. *P. baldaccii* which was described as finely rounded to almost acute [75,77]. *Paratylenchus baldaccii* is described morphologically in this study (Table 6, Figure 14) with molecular markers provided too (see description below) and different to other molecular species descriptions within the *P. hamatus* “species complex” [23]. Our population of *P. baldaccii* matches with the original description of the species, but in our samples it is really very difficult to separate *P. baldaccii* and *P. hamatus* only based on morphological traits. However, topotypes of *P. baldaccii* should be necessary in order to assign a definitive molecular marker association between morphology and molecular differences in this complex species group. Specimens of *P. hamatus* from fig orchards at the type locality (Planada, CA, USA) were molecularly characterized by Van den Berg et al. [23]. *Paratylenchus hamatus* has not been described before in Spain and constitute a first record for the country.

**Figure 15:**
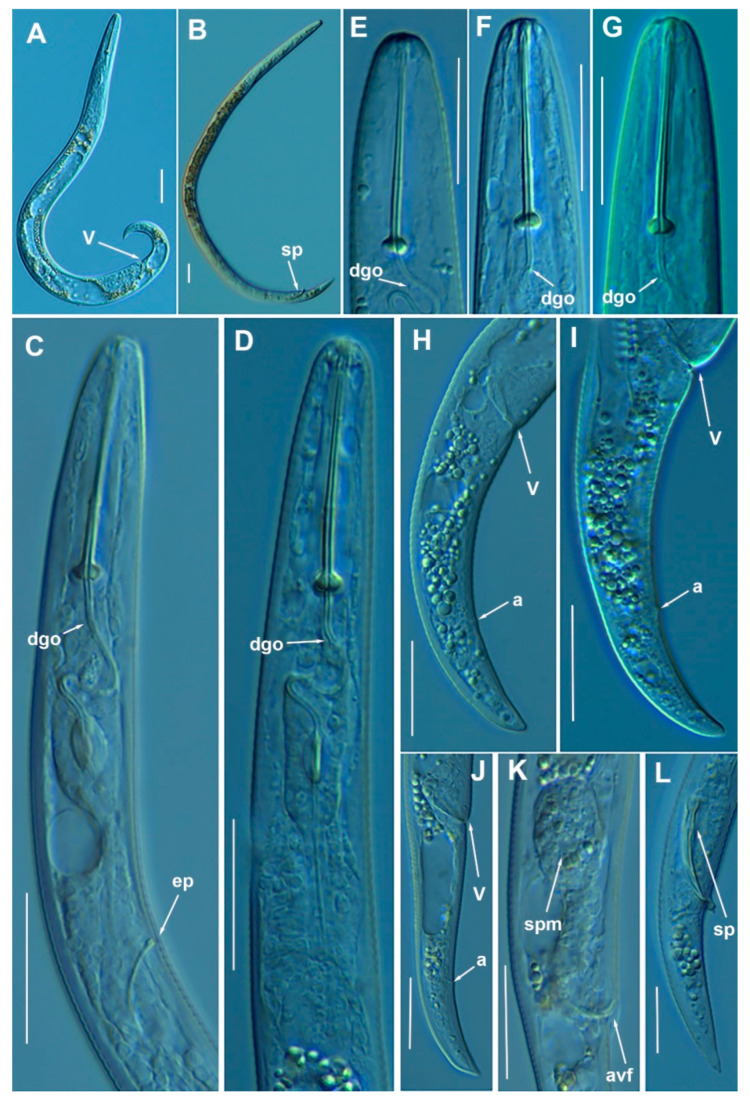
Light photomicrographs of *Paratylenchus hamatus* Thorne & Allen, 1950. (**A**): Entire female with vulva arrowed; (**B**): Entire male with spicules arrowed; (**C**,**D)**: Female pharyngeal region; (**E**–**G**): Female lip region; (**H**–**J**): Female posterior region with vulva and anus (arrowed); (**K**): Detail of vulva showing spermatheca and advulval flap (arrowed); (**L**): Male tail with spicules arrowed. Scale bars (**A**–**L** = 20 μm). (Abbreviations: a = anus; avf = advulval flap; dgo = pharyngeal dorsal gland orifice; ep = excretory pore; spm = spermatheca; sp = spicules; V = vulva).

*Paratylenchus hamatus* has a worldwide distribution and has been reported in many countries including Australia, Belgium, Canada, Pakistan, etc. [36]. This species can cause damage in different crops as figs and several vegetables [36]. Different populations of this species have been found in our study in the rhizosphere of peach/rootstock peach × almond [GxN] (sample codes PR_44 and PR_207) and almond/rootstock almond orchards (sample codes PR_115 and PR_187). Nematode soil population levels were high or very high (42400, 4212, 2042 and 8250 individuals per 500 cm^3^ of soil) in peach orchards sample codes PR_44, PR_115, PR_187 and PR_207, respectively. However, peach trees did not show any apparent growth reduction or symptomatology associated with these high levels of nematodes in the soil. Morphologically, the Spanish populations studied herein (Figure 15, Table 7) were in the range of the described populations and topotypes from *P. hamatus* [23].

*Paratylenchus baldaccii* has been described in grapevine in Sicily (Italy), Bari (Italy) and South of France [77] and later in Italy [75] and Spain [44]. In our sampling has been recorded in one locality in a peach orchard at a density of 200 individuals per 500 cc of soil.

*Molecular characterization*: Five D2-D3 of 28S rRNA (MW798295- MW798299), two ITS (MW798340-MW798341), and two COI gene sequences (MW797016-MW797017) of *P. hamatus* were generated in this study without intraspecific sequence variations. All of sequences were found to be, respectively, identical to KF242208, KF242248 and MN711355, accessions belonging to *P. hamatus* from USA [23,78].

For *P. baldaccii*, two D2-D3 of 28S rRNA (MW798290-MW798291), two ITS (MW798335-MW798336), and one COI gene sequences (MW797012) were generated herein without intraspecific sequence variations. The closest *Paratylenchus* sequences to *P. baldaccii* were those of *P. pedrami* sp. nov. with 96, 93 and 90% similarity (differing by 30, 45 and 26 nucleotides) for the D2-D3 of 28S rRNA (MW798283-MW798285), ITS region (MW798329-MW798330) and COI gene (MW797009), respectively.

*Paratylenchus holdemani* Raski, 1975.

**Figure 16:**
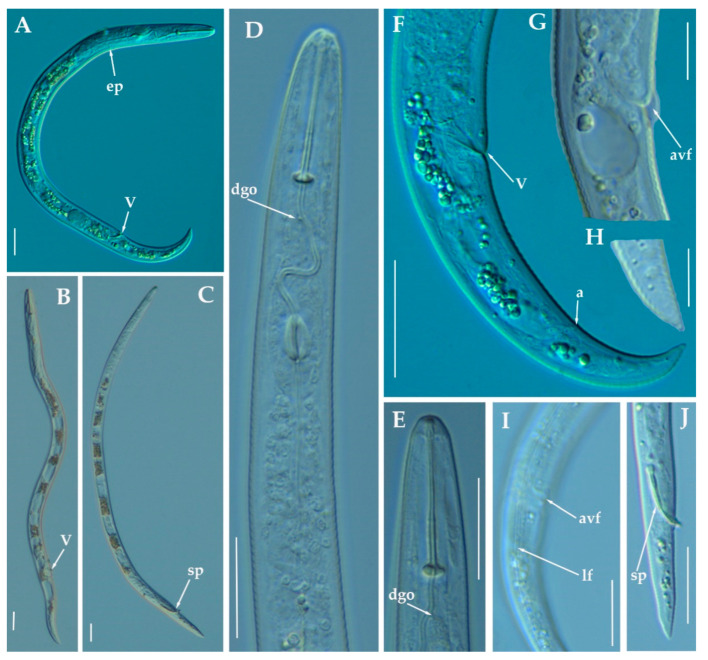
Light photomicrographs of *Paratylenchus holdemani* Raski, 1975. (**A**,**B**): Entire female with vulva arrowed; (**C**): Entire male with spicules arrowed; (**D**): Female pharyngeal region; E: Female lip region; F: Female posterior region with vulva and anus (arrowed); (**G**): Detail of vulva showing advulval flap (arrowed); (**H**): Detail of female tail tip; (**I**): Female posterior region showing lateral field and advulval flap (arrowed); (**J**): Male tail with spicules arrowed. Scale bars (**A**–**F**,**I**,**J** = 20 μm; **G**–**H** = 10 μm). (Abbreviations: a= anus; avf= advulval flap; dgo= pharyngeal dorsal gland orifice; ep = excretory pore; lf = lateral field; sp = spicules; V = vulva).

This species has been described from Santa Ana, El Salvador [77] and also reported in Czech Republic [33] and Belgium [24]. It resembles morphologically *P. hamatus* and *P. baldaccii* but differs from them in having a shorter female stylet [36]. The morphology and morphometry of the Spanish population from Martos, Jaén province (Southern Spain) (Table 6, Figure 16) agrees with the original species description and other populations described, as well as molecularly in various molecular markers to populations sequenced for this species [24]. Minor differences were found in longer female body (345–441 µm vs. 290–350 µm), higher a ratio (23.5–27.2 vs. 19–24) and longer stylet (24–29 µm vs. 21–23 µm) in comparison to paratypes. However, later species descriptions increased the overlapping ranges for some important characters as longer body (285–475 µm), longer stylet (19.0–26.1 µm) [24]. In our case the morphometrics and molecular data were coincident with Singh et al. [24] and increase the morphological traits range of the species even further than the original description of the species. The presence of males is also reported in the Spanish population. The integrative taxonomical identification of this population confirms the morphometrical plasticity of this species. Only one population has been found in an almond orchard in Martos, Jaén province with 4735 individuals per 500 cm^3^ of soil. This species has not been described before in Spain and constitutes a first report for the country and expand their distribution in Europe.

*Molecular characterization*: One sequence from all regions were generated in this study, MW798300, MW798342 and MW797018 (D2-D3 of 28S rRNA, ITS and COI gene, respectively) being all of them identical to several accessions from *P. holdemani* deposited in GenBank, such as MW413642 for the D2-D3 of 28S, MW413596 for the ITS region and MW421652 for the COI gene [24].

*Paratylenchus israelensis* (Raski, 1973) Siddiqi, 1986.

**Figure 17:**
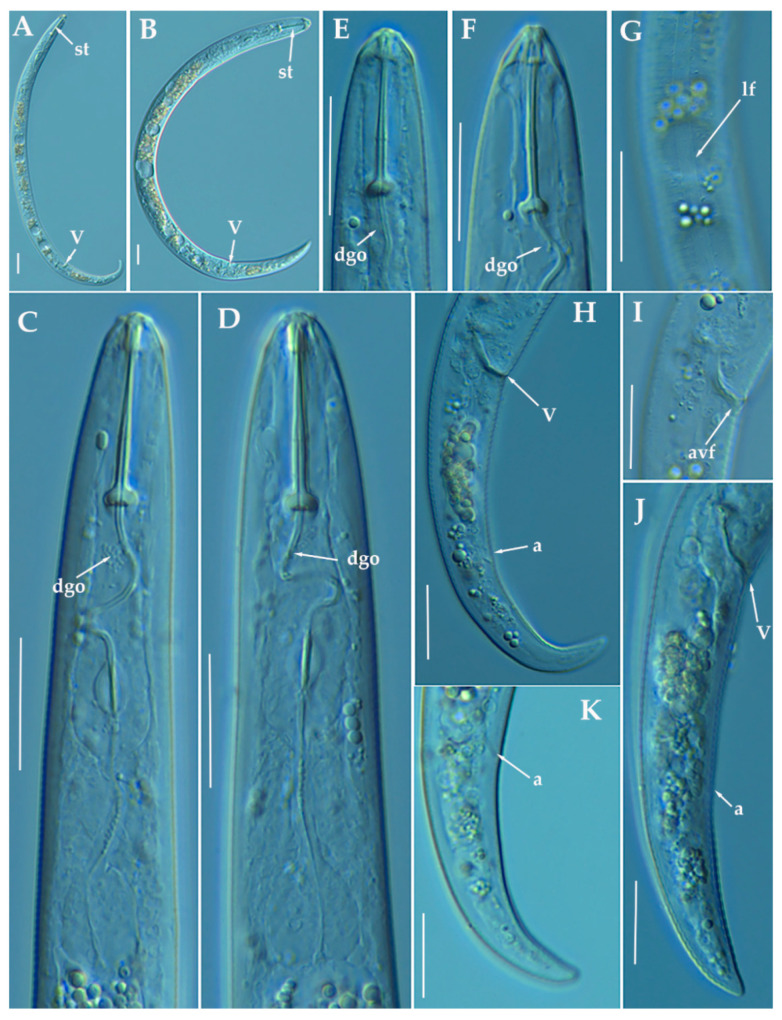
Light photomicrographs of *Paratylenchus israelensis* (Raski, 1973) Siddiqi, 1986. (**A**,**B**): Entire female with vulva arrowed; (**C**,**D**): Female pharyngeal region; (**E**,**F**): Female lip region; (**G**): Detail of lateral field at mid-body; (**H**,**J**): Female posterior region with vulva and anus (arrowed); (**I**): Detail of vulva showing advulval flap (arrowed); (**K**): Detail of female tail tip with anus arrowed. Scale bars (**A**–**K** = 20 μm). (Abbreviations: a = anus; avf = advulval flap; dgo = pharyngeal dorsal gland orifice; lf = lateral field; V = vulva).

This species was described in Shiller, Israel [79]. It is characterized by a strong sclerotization of the lip region. This species is similar to *P. sheri*, from which differs in its longer more robust stylet, stronger sclerotization of the lip region and different outline of lateral field in cross section [36]. The Spanish populations from two localities in Córdoba province (Southern Spain) fit the original description of *P. israelensis* and no differences were found (Table 6, Figure 17). Soil populations from almond orchards in Córdoba (sample code PR_011) and in Valenzuela (sample code PR_079) showed 368 and 320 individuals/500 cm^3^ of soil, respectively. No symptoms of decline were apparently detected in the trees. This species has not been described before in Spain and constitute a first record for the country.

*Molecular characterization*: Five D2-D3 of 28S rRNA (MW798301-MW798305), four ITS (MW798343-MW798346) and two COI (MW797019-MW797020) gene sequences were generated for the first time from this species without intraspecific sequence variations, except for the ITS sequences (differing by 1 nucleotide and 2 indels). The closest *Paratylenchus* spp. was *P. neoamblycephalus* described in Singh et al. [24] with 99% similarity for the D2-D3 of 28S rRNA (differing by 7 nucleotides) to MW413660-MW413663, for the ITS sequences the similarity was 95% (differing by 45 nucleotides and 13 indels) with MW413606-MW413609, and finally sequences from COI gene regions showed a similarity of 90% (37 nucleotides) with the accession (MW421675-MW421682). However, morphologically and morphometrically *P. israelensis* and *P. neoamblycephalus* can be clearly separated by: labial framework (with strong sclerotization vs. light sclerotization), lip region shape (conical with protruding submedian lobes surrounding the oral aperture vs. conical-truncate with submedian lobes indistinct), and stylet length (24–26 μm vs. 26–34 μm) [36].

*Paratylenchus tenuicaudatus* Wu, 1961.

Described from Ontario, Canada in soil around roots of *T. pratense*, *T. repens* L., *Acer saccharum* Marsh, *M. sativa*, and grass sod. It has also been reported in several localities of USA [77,80] and Iran [81]. The Iranian population of *P. tenuicaudatus* was also morphologically and molecularly similar to *Paratylenchus* sp. 1 from USA [23], suggesting that *Paratylenchus* sp. 1 from USA is conspecific with *P. tenuicaudatus* as mentioned by Esmaeili et al. [81].

**Figure 18:**
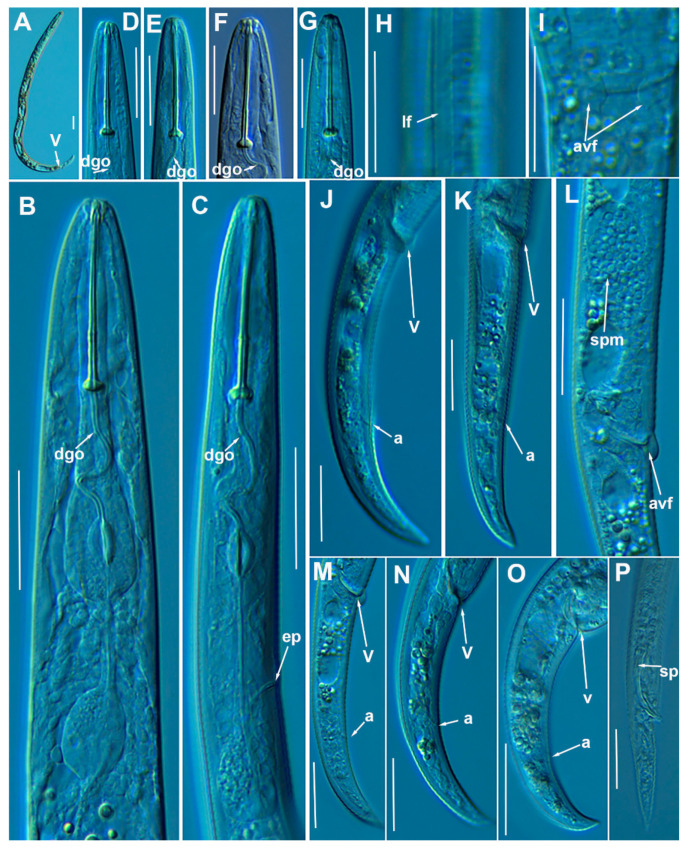
Light photomicrographs of *Paratylenchus tenuicaudatus* Wu, 1961. (**A**): Entire female with vulva arrowed; (**B**,**C**): Female pharyngeal region; (**D**–**G**): Female lip region; (**H**): Detail of female lateral field at mid-body (arrowed); (**I**): Detail of vulva showing a frontal view of advulval flap (arrowed); (**J**–**O**): Female posterior region showing vulva and anus (arrowed); (**P**): Male tail with spicules arrowed. Scale bars (**A**–**P** = 20 μm). (Abbreviations: a = anus; avf = advulval flap; dgo = pharyngeal dorsal gland orifice; ep = excretory pore; lf = lateral field; spm = spermatheca; sp= spicules; V = vulva).

The four Spanish populations of *P. tenuicaudatus* detected in our study agree with original description and others populations with molecular data available (Table 8, Figure 18). Only minor differences were found in all populations measured in a slightly shorter female body in comparison to the original description (354–392 µm, 307–407 µm, 292–389 µm, and 394–414 µm vs. 381–600 µm), but with a good size matching with the Iranian population (305–365 µm); slightly posterior positon of the vulva in the Caravaca population (sample code PR_124) (82.1–84.7% vs. 77.6–81.6%) and longer female stylet in Sástago population (sample code PR_208) (32.0–33.5 µm vs. 25.1–31.5 µm). Molecularly the Spanish populations are identical to the populations with molecular data available from Iran [81] and USA population (identified as *Paratylenchus* sp. 1) [23]. These four populations have been found in the rhizosphere of almond in Caravaca, Murcia province (sample code PR_124), peach in Calasparra, Murcia province (sample code PR_129), peach in Sollana, Valencia province (sample code PR_168) and apricot in Sástago, Zaragoza province (sample code PR_208) with 212, 10149, 15.050 and 12.950 individuals/500 cm^3^ of soil, respectively. This species has been previously reported in Navarra forests (North Spain) by Hernández et al. [52], but no detailed morphology and measurements were provided.

*Molecular characterization*: Four D2-D3 of 28S rRNA (MW798306-MW798309), three ITS (MW798347-MW798349), and three COI gene sequences (MW797021-MW797023) were obtained from this species without intraspecific sequence variations. COI sequences were generated the first time in this study. D2-D3 of 28S rRNA sequences were shown to be 99% similar to the accession KU291239 (differing by 4 nucleotides) and ITS sequences were identical to the accession KF242260 [24,81].

*Paratylenchus veruculatus* Wu, 1962.

Described from soil around roots of heather from Kilmcolm, Scotland [82]. It has been also reported in Scotland [83], Belgium [24,83], Russia [73], Poland [33,75] and Iran [84].

**Figure 19:**
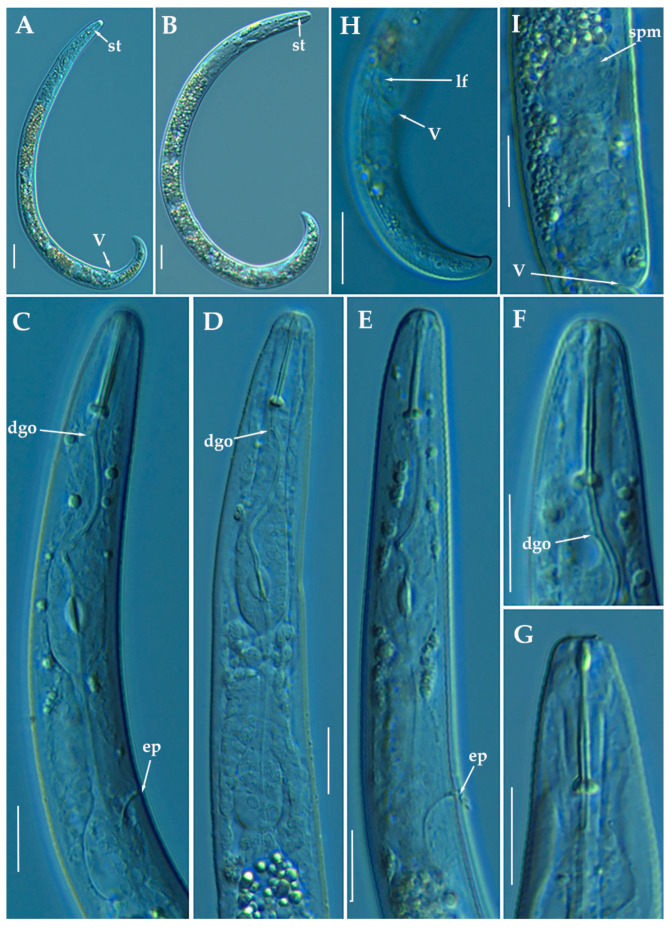
Light photomicrographs of female of *Paratylenchus veruculatus* Wu, 1962. (**A**): Entire female with stylet and vulva arrowed; (**B**): Fourth-stage juvenile with stylet arrowed; (**C**–**E**): Female pharyngeal region; (**F**–**G**): Female lip region; (**H**): Female posterior region with lateral field and vulva arrowed; (**I**): Detail of empty spermatheca (arrowed). Scale bars (**A**,**B**,**H** = 20 μm; **C**–**G**,**I**= 10 μm). (Abbreviations: a = anus; dgo = pharyngeal dorsal gland orifice; ep = excretory pore; lf = lateral field; spm = spermatheca; st = stylet; V = vulva).

The five Spanish populations of *P. veruculatus* agree with the original species description and other populations [24,75,83,84], at exception of longer body in comparison to the original description (354–436 µm, 349–407 µm, 303–445 µm, 353–395 µm, 279–441 µm vs. 250–320 µm) and the other four populations of this species cited in the literature (270 µm, 230–320 µm, 290–350 µm, and 251–331 µm) (Table 9, Figure 19). Longer stylet in comparison to the original description (14–16 µm, 15.5–16.0 µm, 14.5–16.5 µm, 15.5–16.0 µm, 15.0–17.0 µm vs. 12.0–15.0 µm) and others (14 µm, 10.5–14.5 µm, 11.0–14.0 µm, and 13.1–14.8 µm). However, all molecular markers of the Spanish populations agree with their identification as *P. veruculatus* with the molecular markers provided by Singh et al. [24]. These five populations have been found in almond in Puebla de Don Fadrique, Granada province (sample code PR_122), almond in Sta. Mª de Nieva, Almería province (sample code PR_106), almond in Lúcar, Almería province (sample code PR_115), almond in Serón, Almería province (sample code PR_118) and peach in La Almunia, Zaragoza province (sample code PR_193) with 180, 630, 1404, 1134 and 20 individuals/500 cm^3^ of soil, respectively. This species has not been reported before in Spain and constitute a first record for the country.

*Molecular characterization*: Six D2-D3 of 28S rRNA (MW798310-MW798315) with a intraspecific sequence variation of 2% (differing from 0 to 13 nucleotides and 1 indel), five ITS (MW798350-MW798354) (96% similarity; 27 nucleotides and 6 indels), and finally, six COI gene sequences (MW797024-MW797029) with a intraspecific sequence variation of 5% (differing from 0 to 19 nucleotides). The D2-D3 of 28S rRNA sequences matched well with other accession of *P. veruculatus* deposited in GenBank showing similarity values from 98 to 99% (differing from 1 to 10 nucleotides and 1 indel) with MW413687, and from 94 to 95% for the COI gene sequences (differing from 19 to 21 nucleotides) with MW421717 [24]. ITS sequences (MW798350-MW798354) from *P. veruculatus* were generated for the first time in this study.

### 3.2. Phylogenetic Analyses

**Figure 20:**
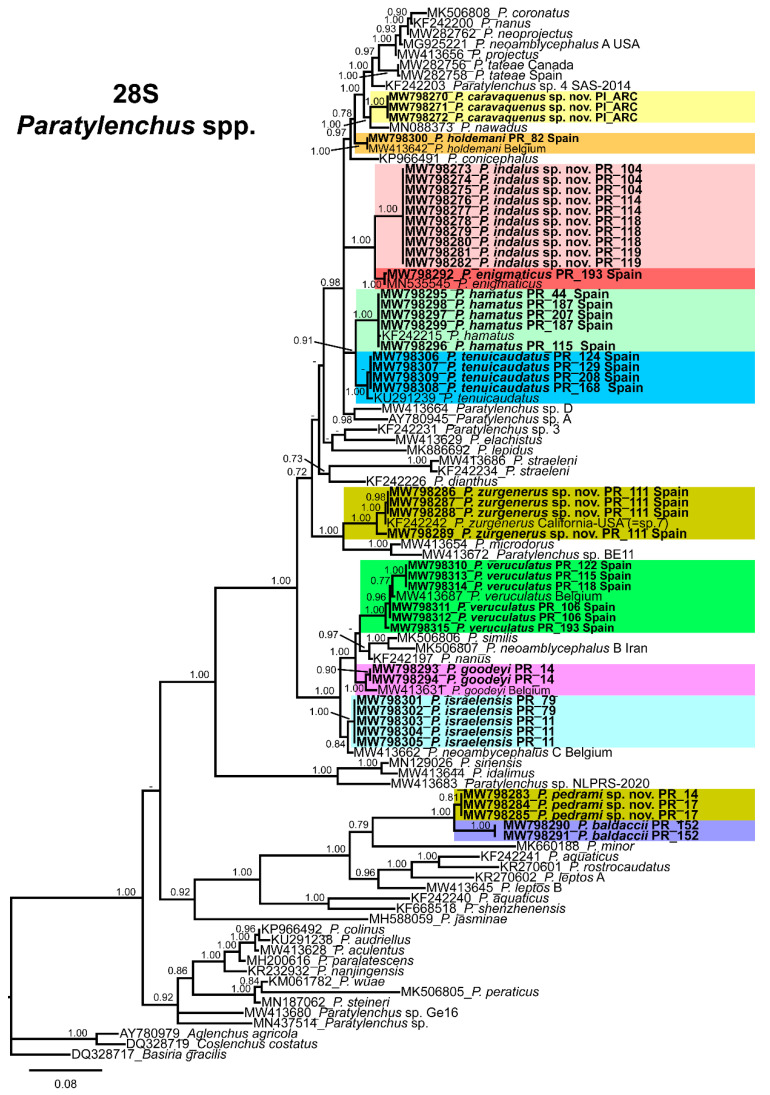
Phylogenetic relationships within the genus *Paratylenchus*. Bayesian 50% majority rule consensus tree as inferred from D2-D3 expansion domains of the 28S rRNA sequence alignment under the general time-reversible model of sequence evolution with correction for invariable sites and a gamma-shaped distribution (GTR + I + G). Posterior probabilities of more than 0.70 are given for appropriate clades. Newly obtained sequences in this study are shown in bold. The scale bar indicates expected changes per site.

The D2-D3 domains of the 28S rRNA gene alignment (699 bp long) included 98 sequences of 54 *Paratylenchus* species and three outgroup species [*Basiria gracillis* (DQ328717), *Aglenchus agricola* (AY780979), and *Coslenchus costatus* (DQ328719)]. Forty-six new sequences were included in this analysis. The Bayesian 50% majority rule consensus tree inferred from the D2-D3 alignment is given in Figure 20. The tree contained one highly supported major clade (PP = 1.0) and two moderately supported clades (PP = 0.92, PP = 0.92)). These clades are mainly coincident with other recent studies on *Paratylenchus* spp. [24,28].

**Figure 21:**
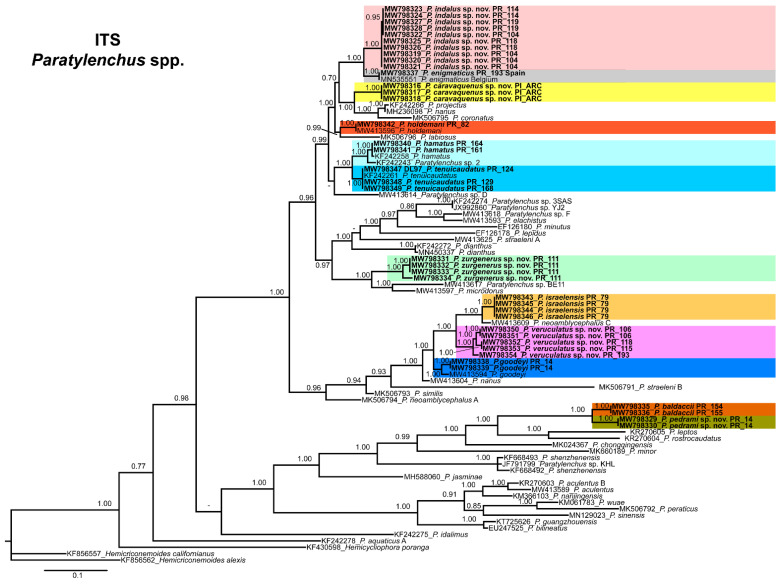
Phylogenetic relationships within the genus *Paratylenchus*. Bayesian 50% majority rule consensus tree as inferred from ITS rRNA sequence alignment under the general time-reversible model of sequence evolution with correction for invariable sites and a gamma-shaped distribution (GTR + I + G). Posterior probabilities of more than 0.70 are given for appropriate clades. Newly obtained sequences in this study are shown in bold. The scale bar indicates expected changes per site.

The ITS rRNA gene alignment (778 bp long) included 84 sequences of 48 *Paratylenchus* species and three outgroup species [*Hemicriconemoides californianus* (KF856557), *Hemicriconemoides alexis* (KF856562) and *Hemicycliophora poranga* (KF430598)]. Thirty-seven new sequences were included in this analysis. The Bayesian 50% majority rule consensus tree inferred from the ITS alignment is given in Figure 21. The tree contained one highly supported major clade (PP = 1.00) and other clade not supported (but highly supported if excluding *P. idalimus* (KF242275)). These clades were partially coincident with previous studies with in some case similar or different clade support [24,28].

**Figure 22:**
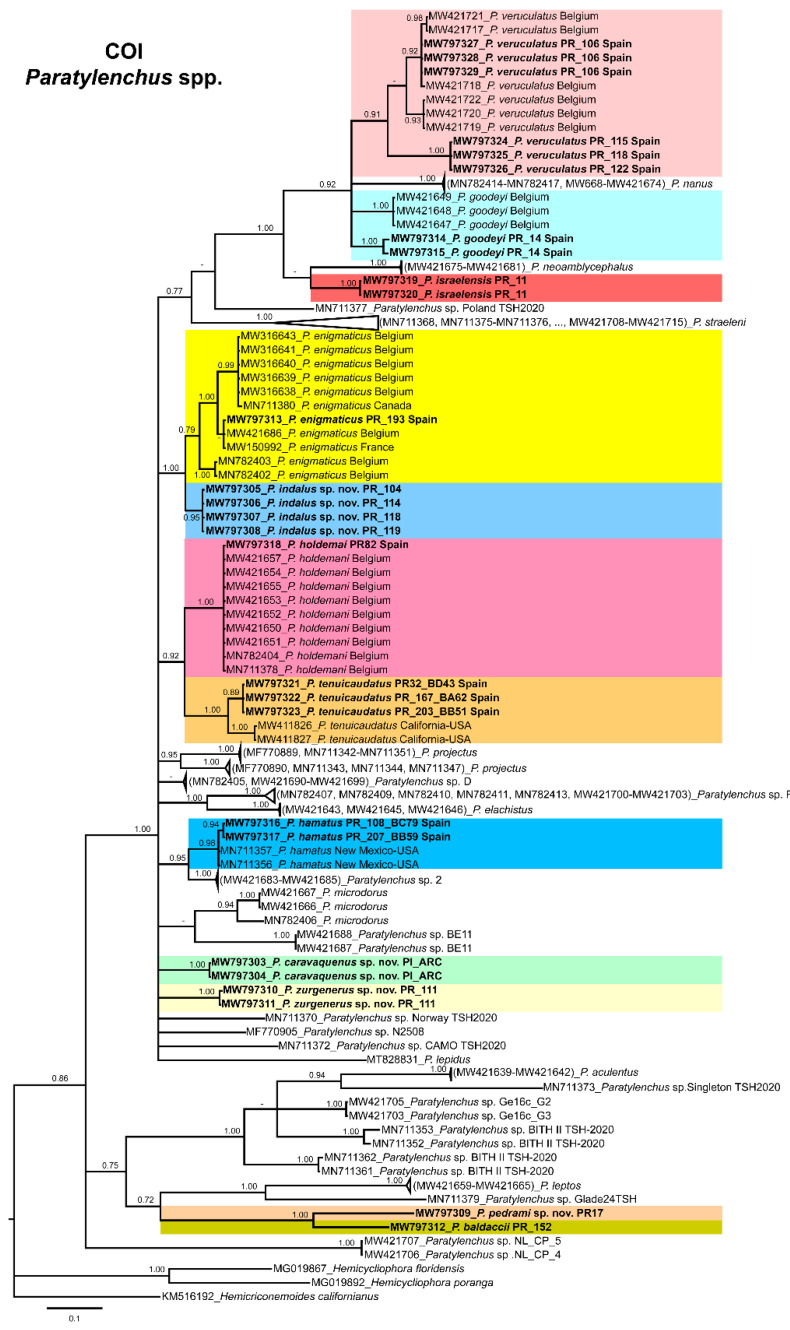
Phylogenetic relationships within the genus *Paratylenchus*. Bayesian 50% majority rule consensus tree as inferred from COI sequence alignment under the general time-reversible model of sequence evolution with a gamma-shaped distribution (GTR + G). Posterior probabilities of more than 0.70 are given for appropriate clades. Newly obtained sequences in this study are shown in bold. The scale bar indicates expected changes per site.

The COI gene alignment (340 bp long) included 161 sequences of 34 *Paratylenchus* species and three outgroup species [*Hemicriconemoides californianus* (KM516192), *Hemicycliophora floridensis* (MG019867) and *Hemicycliophora poranga* (MG019892)]. Seventy-one new sequences were included in this analysis. The Bayesian 50% majority rule consensus tree inferred from the COI sequence alignment is given in Figure 22. The tree contained one highly supported major clade (PP = 1.00) and one moderately supported clade (PP = 0.75). These clades were partially coincident with other studies with in some case similar or different clade support [24].

*Paratylenchus caravaquenus* sp. nov. is closely related phylogenetically in the D2-D3 domains of the 28S rRNA to *P. nawadus* (MN088373) in a high supported clade (PP = 1.00). These two species are related in a larger clade to *Paratylenchus* sp. 4 SAS-2014 (KF242203), *P. tateae* (MW282756 and MW282758), *P. projectus* (MW413656), *P. neoamblycephalus* A USA (MG925221), *P. neoprojectus* (MW282762), *P. nanus* (KF242200) and *P. coronatus* (MK506808) (PP = 1.00). For ITS region, *P. caravaquenus* sp. nov. is related with *P. projectus* (KF242266), *P. nanus* (MH236098) and *P. coronatus* (MK506795) in a high supported clade (PP = 1.00) (Figure 21). While for COI marker, this species was separated in a clade, but their phylogenetic relationship was not clearly defined with other species for this marker (Figure 22).

*Paratylenchus indalus* sp. nov. was closely related in all markers studied (D2-D3 domains of the 28S rRNA, ITS and COI) with *P. enigmaticus* with high-supported clades (PP = 0.98–1.00) (Figure 20, Figure 21 and Figure 22).

*Paratylenchus pedrami* sp. nov. was closely related in all markers studied (D2-D3 domains of the 28S rRNA, ITS and COI) with *P. baldaccii* with high-supported clades (PP = 1.00) (Figure 20, Figure 21 and Figure 22).

*Paratylenchus zurgenerus* sp. nov. was closely related to *Paratylenchus* sp. 7 SAS-2014 (KF242242) in D2-D3 tree (most probably is conspecific with *P. zurgenerus* sp. nov.) and to *P. microdorus* (MW413654) and an undescribed species *Paratylenchus* sp. BE11 (MW413672), and in ITS tree also related to *P. microdorus* (MW413597) and an undescribed species *Paratylenchus* sp. BE11 (MW413617) (Figure 20 and Figure 21). COI marker did not show a clear relationship with other species, but this species was clearly separated from the other species in a unique clade (Figure 22).

Species identification with molecular markers deposited in GenBank was completely congruent with their phylogenetic position as it the case for *P. hamatus*, *P. tenuicaudatus*, *P. holdemani*, *P. enigmaticus*, *P. veruculatus*, and *P. goodeyi* (Figure 20, Figure 21 and Figure 22). Species with new molecular information but already described in the literature include *P. israelensis* and *P. baldaccii*. *Paratylenchus israelensis* is closely related to *P. neoamblycephalus* C Belgium (MW413662) in a moderately supported clade (PP = 0.84) in the D2-D3 domains of the 28S rRNA, in a high-supported clade (PP = 1.00) in the ITS region (MW413609), and a low-supported clade (PP < 0.70) in the COI tree (MW421675-MW421681) (Figure 20, Figure 21 and Figure 22). *Paratylenchus baldaccii* is closely related phylogenetically with *P. pedrami* sp. nov. as described before (Figure 20, Figure 21 and Figure 22).

## 4. Discussion

This research is the major study of pin nematodes of the genus *Paratylenchus* carried out in Spain, increasing their biodiversity, confirming the great cryptic morphology among several species, and expanding the number of species of this genus with molecular information for their unequivocal identification. Herein, we provide detailed morphological and molecular data on 27 studied populations from Spain and also discuss and confirm the existence of cryptic species as suggested in other plant-parasitic nematodes [85,86]. More specifically, four new species were described and additionally of them, five out of eight species identified are considered first reports in Spain (*P. enigmaticus*, *P. hamatus*, *P. holdemani*, *P. israelensis*, and *P. veruculatus*). This study also confirms that we have only found just a minor part of the species was already reported in Spain as *P. baldaccii, P. goodeyi* and *P. tenuicaudatus* [44,48,52], indicating that the biodiversity of this group is far to be adequately explored in Spain and many of the data need to be revisited and complemented with molecular tools for their accurate identification by integrative taxonomy. However, our data in the present research were mainly based on agricultural systems and some of these species previously reported in Spain may not be coincident with our present species found mainly in *Prunus* plantations. Some of our data, even for new species, reinforces the idea that some of these species might be pathogenic in some periods of the year or feed with other plant hosts different from the main crop observed at the time of the soil sampling. This idea is reinforced because the majority of the species described here are in an arrested juvenile stage at the moment of sampling. Additionally, many ecological requirements of these nematodes are fairly deciphered (degree of soil humidity, temperature, etc.), and further studies are required for clarifying these aspects.

This study gave molecular markers for the first time for several *Paratylenchus* species for their accurate identification in an integrative taxonomic approach (including molecular and morphological traits). This is even more important where the presence of cryptic speciation is clearly detected in this genus (as for example, *P. aquaticus, P. straeleni* or *P. hamatus* [23,24]), with an excellent new example described in this study (i.e., *P. indalus* sp. nov., *P. projectus, P. neoprojectus* and *P. enigmaticus*). Several authors studied and give molecular markers in an integrative approach for this genus [21,23,24,26,27,28,29]. Singh et al. [24] gives a DNA-based species delimitation study using different calculation approaches and the markers most congruent between species separation by morphology and molecular identification was COI and D2-D3 region of 28S rRNA. These same authors recommend the use of multilocus approaches (D2-D3 region of 28S rRNA and COI) for a posterior double-check for contamination, sequencing errors of mitochondria-specific pitfalls [87]. Our data agrees with this result, but the phylogeny with COI marker was not completely congruent with the ribosomal molecular markers, and also some clades were low-supported. Phylogenetic analyses based on D2-D3, ITS, and partial COI gene using BI resulted in a consistent position for the newly described species of *Paratylenchus* species from Spain, and mostly agree with the clustering obtained by other authors [24,28]. Although the position of some species varies, probably due to the large number of species and additional molecular diversity included in this study. The molecular markers for this genus match with our identified species as mentioned in results, giving evidence that they could help in the identification process for the majority of our species. In our study, the majority of the species showed no or low intraspecific molecular variability for ribosomal and mitochondrial regions irrespective of the geographic origin of the population, with illustrative examples in *P. indalus* sp. nov., *P. goodeyi* or *P. hamatus*. The highest intraspecific variability for COI and ITS regions (95%, 96%, respectively) was found in *P. veruculatus*. Singh et al. [24] detected also important COI gene sequence variations within some species such as *P. enigmaticus*, *P. microdorus* and *P. veruculatus*, despite these sequences were originated from the same population. Usually, the most variable marker for plant-parasitic nematode species separation is COI, followed by ITS, D2-D3 region of 28S rRNA and partial 18S rRNA [85,88]. However, in this study we found a different molecular variability among the different molecular markers between our four populations of *P. indalus* sp. nov. and molecular markers for *P. enigmaticus* in Canada [21] and Belgium [24]. In this case, the variability is higher or similar for the D2-D3 region of 28S rRNA marker than for ITS region and COI (96% vs. 94–96% and 97% similarity for D2-D3 region of 28S rRNA, ITS region and COI, respectively when compared both species). This point is reinforced because many sequences were obtained for *P. indalus* sp. nov. (ten D2-D3 of 28S rRNA, thirteen ITS, and four COI gene sequences) from the four different populations and all populations showed no intraspecific variation, except for the ITS sequences, where one variable position was found. This result is difficult to explain, and reinforces the idea that more than one marker is necessary for the molecularly species identification in this group of nematodes. In this case, the low molecular differences for COI marker needs to be explored in further studies with different mitochondrial markers or different primers for this gene.

Interestingly, the morphology in some complex species did not match the phylogenetic results, for example, the “*P. hamatus* complex” of species has some species closely related phylogenetically, but other far related such as *P. pedrami* sp. nov. and *P. baldaccii*, but these later related morphologically. Clear morphological characters did not match our phylogenies, for example the long stylet length (>40 μm) for some species did not correspond with a unique clade (*P. straeleni*, *P. goodeyi*, *P. idalimus*.) as this character seems evolved independently several times in the different phylogenetic trees. Two clades of species seem to evolved from an exclusive long stylet ancestor, for example clade II (*P. idalimus*, *P. sinensis* and others) or the subclade (*P. colinus*, *P. audriellus*, *P. aculentus*, *P. paralatescens*, *P. nanjingensis* and *P. peraticus*) in the D2-D3 region of 28S rRNA tree, but not clearly supported in the other trees in this study or only partially in specific subclades (ITS region and COI). In this sense, the use of fresh material is essential to observe several characters in these nematodes as the presence/absence of stylet in male and juveniles, advulval flaps, lateral field incisures, and other characters difficult to visualize in the glycerin mounted specimens.

The distribution of many of these *Paratylenchus* species using an integrative approach showed that these species are more widely spread among continents that suspected. Cases as *P. hamatus*, *P. tenuicaudatus*, *P. enigmaticus*, …) shows us that these nematodes have a potential to be distributed on soil remains even with the absence of plants because of their resistant strategies not properly studied. As commented before, the majority of our samples are based on cultivated soils, but probably a higher diversity could be present in samples from wild environments.

## 5. Conclusions

This study describes and provides unequivocal molecular markers for the identification of different *Paratylenchus* species found in Spain. This is particularly important in this group of nematodes as only a few morphological characters can be used in their identification. As pointed in this and other studies, molecular markers could help in their identification, even when morphological characters might be variable and not overlapping ranges can be found. In summary, the present study confirmed the cryptic diversity of *Paratylenchus* species in Spain and comprises a good example of morphostatic speciation of pin nematodes in Spain. However, this genus is started recently to be studied by integrative taxonomy and increasing numbers of examples of cryptic diversity is expected to be found in the future.

## Figures and Tables

**Table 1:** Isolates sampled and sequenced for *Paratylenchus* spp. from several localities in Spain used in this study. Different rootstocks could be used in the cultivated plants.

Soil Sample Code	Nematode Species	Locality (Province)	Host Plant	D2-D3	ITS	COI
PI_ARC	***Paratylenchus caravaquenus* sp. nov.**	Caravaca (Murcia) ^a^	pine	MW798270-MW798272	MW798316-MW798318	MW797003-MW797004
PR_104	***Paratylenchus indalus* sp. nov.**	Santa María de Nieva (Almería) ^a^	almond	MW798273-MW798275	MW798319-MW798322	MW797005
PR_114	***Paratylenchus indalus* sp. nov.**	Urracal (Almería)	almond	MW798276-MW798277	MW798323-MW798324	MW797006
PR_118	***Paratylenchus indalus* sp. nov.**	Serón (Almería)	almond	MW798278-MW798280	MW798325-MW798326	MW797007
PR_119	***Paratylenchus indalus* sp. nov.**	El Hijate (Almería)	almond	MW798281-MW798282	MW798327-MW798328	MW797008
PR_014	***Paratylenchus pedrami* sp. nov.**	Córdoba (Córdoba) ^a^	almond	MW798283	MW798329	MW797009
PR_017	***Paratylenchus pedrami* sp. nov.**	Córdoba (Córdoba)	almond	MW798284-MW798285	MW798330	-
PR_111	***Paratylenchus zurgenerus* sp. nov.**	Zurgena (Almería) ^a^	almond	MW798286-MW798289	MW798331-MW798334	MW797010-MW797011
PR_152	*Paratylenchus baldaccii* Raski, 1975	Cantillana (Sevilla)	peach	MW798290-MW798291	MW798335-MW798336	MW797012
PR_193	*Paratylenchus enigmaticus* Munawar, 2021	La Almunia (Zaragoza)	cherry	MW798292	MW798337	MW797013
PR_014	*Paratylenchus goodeyi* (Oostenbrink, 1953) Raski, 1962	Córdoba (Córdoba)	almond	MW798293-MW798294	MW798338-MW798339	MW797014-MW797015
PR-044	*Paratylenchus hamatus* Thorne & Allen, 1950	Gibraleón (Huelva)	peach	MW798295	MW798340	MW797016
PR_115	*Paratylenchus hamatus* Thorne & Allen, 1950	Lúcar (Almería)	almond	MW798296	MW798341	-
PR_207	*Paratylenchus hamatus* Thorne & Allen, 1950	Sástago (Zaragoza)	peach	MW798297	-	MW797017
PR_187	*Paratylenchus hamatus* Thorne & Allen, 1950	Ariza (Zaragoza)	almond	MW798298-MW798299	-	-
PR_082	*Paratylenchus holdemani* Raski, 1975	Martos (Jaén)	almond	MW798300	MW798342	MW797018
PR_079	*Paratylenchus israelensis* (Raski, 1973) Siddiqi, 1986	Valenzuela (Córdoba)	almond	MW798301-MW798302	MW798343–MW798346	-
PR_011	*Paratylenchus israelensis* (Raski, 1973) Siddiqi, 1986	Córdoba (Córdoba)	almond	MW798303-MW798305	-	MW797019-MW797020
PR_124	*Paratylenchus tenuicaudatus* Wu, 1961	Caravaca (Murcia)	almond	MW798306	MW798347	MW797021
PR_129	*Paratylenchus tenuicaudatus* Wu, 1961	Calasparra (Murcia)	nectarine	MW798307	MW798348	MW797022
PR_168	*Paratylenchus tenuicaudatus* Wu, 1961	Sollana (Valencia)	nectarine	MW798308	MW798349	MW797023
PR_208	*Paratylenchus tenuicaudatus* Wu, 1961	Sástago (Zaragoza)	apricot	MW798309	-	-
PR_122	*Paratylenchus veruculatus* Wu, 1962	El Moral (Murcia)	almond	MW798310	-	MW797026
PR_106	*Paratylenchus veruculatus* Wu, 1962	Santa María de Nieva (Almería)	almond	MW798311-MW798312	MW798350-MW798351	MW797027-MW797029
PR_115	*Paratylenchus veruculatus* Wu, 1962	Lúcar (Almería)	almond	MW798313	MW798352	MW797024
PR_118	*Paratylenchus veruculatus* Wu, 1962	Serón (Almería)	almond	MW798314	MW798353	MW797025
PR_193	*Paratylenchus veruculatus* Wu, 1962	La Almunia (Zaragoza)	cherry	MW798315	MW798354	-

^a^ Type locality (type specimens).

**Table 2:** Morphometrics of *Paratylenchus caravaquenus* sp. nov. paratype females and males. All measurements are in µm and in the form: mean ± s.d. (range).

Measurements and Ratios	Holotype Female	Paratype Females	Paratype Males
Sample code	PI_ARC	PI_ARC	PI_ARC
Locality	Caravaca, Murcia	Caravaca, Murcia	Caravaca, Murcia
n	1	19	6
L	384	384.8 ± 24.4 (344–443)	419 ± 26.0 (372.5–451.5)
a *	24.8	22.6 ± 2.3 (18.4–27.0)	29.2 ± 2.7 (26.6–33.4)
b	3.5	3.7 ± 0.4 (3.2–4.9)	4.4 ± 0.7 (3.7–5.1)
c	15.7	13.9 ± 1.0 (11.2–16.2)	10.96 ± 0.5 (10.5–11.7)
c’	2.7	2.8 ± 0.2 (2.5–3.2)	3.6 ± 0.2 (3.3–3.9)
V or T	83.6	83.4 ± 0.7 (81.8–84.5)	49.5
G1	33.6	36.0 ± 4.5 (29.7–49.0)	-
Stylet length	28.0	29.8 ± 1.5 (26.5–32)	-
Conus length	18.0	19.5 ± 1.5 (16.5–21)	-
m	64.3	65.3 ± 2.6 (60.0–70.0)	-
DGO	7.5	6.8 ± 0.8 (5.5–8)	-
O	26.8	22.9 ± 2.9 (17.2–30.2)	-
Lip width	6.0	6.1 ± 0.5 (5.5–7.0)	4.7 ± 0.3 (4.5–5.0)
Median bulb length	23.0	22.1 ± 3.5 (18.0–29.0)	-
Median bulb width	9.0	10.1 ± 1.1 (9.0–13.5)	-
Anterior end to center median bulb	64.5	60.5 ± 5.5 (47.0–72.0)	50.5
MB	58.6	57.9 ± 2.9 (52.2–64.4)	-
Nerve ring to anterior end	84.0	80.9 ± 7.8 (65.0–96.0)	-
Excretory pore to anterior end	90.0	86.9 ± 4.8 (77.0–98.0)	73.0
Pharynx length	110.0	104.7 ± 8.6 (89.0–124.0)	94.8 ± 5.8 (88.8–100)
Maximum body diam.	15.5	17.2 ± 1.9 (14.5–21.0)	14.5 ± 1.2 (13.5–16.0)
Tail length	24.5	27.9 ± 2.3 (25.0–32.0)	38.3 ± 1.9 (35.5–40.5)
Anal body diam.	9.0	10.1 ± 0.6 (9.0–11.0)	10.7 ± 0.8 (9.5–11.5)
Spicules	-	-	23.3 ± 0.5 (22.5–24.0)
Gubernaculum	-	-	5.3 ± 0.4 (5.0–6.0)

* Abbreviations: a = body length/greatest body diameter; b = body length/distance from anterior end to pharyngo-intestinal junction; DGO = distance between stylet base and orifice of dorsal pharyngeal gland; c = body length/tail length; c’ = tail length/tail diameter at anus or cloaca; G1 = anterior genital branch length expressed as percentage (%) of the body length; L = overall body length; m = length of conus as percentage of total stylet length; MB = distance between anterior end of body and center of median pharyngeal bulb expressed as percentage (%) of the pharynx length; n = number of specimens on which measurements are based; O = DGO as percentage of stylet length; T = distance from cloacal aperture to anterior end of testis expressed as percentage (%) of the body length; V = distance from body anterior end to vulva expressed as percentage (%) of the body length.

**Table 3:** Morphometrics of *Paratylenchus indalus* sp. nov. paratype females and other populations from Spain. All measurements are in µm and in the form: mean ± s.d. (range).

Measurements and Ratios	Holotype Female	Paratype Females	Females	Females	Females
Sample code	PR_104	PR_104	PR_114	PR_118	PR_119
Locality	Sta. Mª Nieva, Almería	Sta. Mª Nieva, Almería	Urracal, Almería	Serón, Almería	El Hijate, Almería
n	1	13	4	2	3
L	377	377.8 ± 36.3 (317–434)	450.5 ± 47.9 (382–484)	370, 399	385 ± 26.2 (357–409)
a *	22.2	19.4 ± 2.3 (16.0–23.7)	24.0 ± 2.3 (21.2–26.8)	19.5, 20.6	20.7 ± 1.9 (19.5–22.9)
b	3.9	3.7 ± 0.3 (3.3–4.2)	4.5 ± 0.3 (4.1–4.7)	3.6, 3.8	3.7 ± 0.2 (3.4–3.9)
c	12.6	12.0 ± 1.6 (9.0–14.8)	16.0 ± 1.9 (13.2–17.3)	13.0, 13.8	12.5 ± 1.2 (11.2–13.4)
c’	3.0	3.0 ± 0.1 (2.7–3.1)	3.0 ± 0.2 (2.8–3.2)	2.8, 2.9	3.0 ± 0.2 (2.9–3.2)
V	83.0	82.9 ± 1.4 (80.4–84.5)	81.6 ± 1.1 (80.6–83.0)	82.4, 82.7	83.4 ± 0.6 (83.0–84.1)
G1	20.4	25.0 ± 4.9 (20.2–38.8)	33.5 ± 7.6 (28.3–44.5)	37.0, 39.8	29.2 ± 7.7 (24.6–38.0)
Stylet length	30.0	28.3 ± 0.9 (26.0–29.5)	31.0 ± 1.4 (29.0–32.0)	28.5, 29.0	28.8 ± 1.0 (28.0–30.0)
Conus length	19.0	18.3 ± 0.6 (17.0–19.0)	18.1 ± 0.6 (17.5–19.0)	17.5, 19.0	18.7 ± 1.2 (18.0–20.0)
m	63.3	64.5 ± 1.6 (62.1–66.7)	58.6 ± 4.8 (54.7–65.5)	61.4, 65.5	64.7 ± 1.8 (63.2–66.7)
DGO	6.5	6.0 ± 0.4 (5.5–6.5)	6.3 ± 0.3 (6.0–6.5)	6.5, 6.5	5.5 ± 0.0 (5.5–5.5)
O	21.7	21.1 ± 1.7 (18.6–23.2)	20.2 ± 1.6 (18.8–22.4)	22.4, 22.8	19.5 ± 0.2 (19.3–19.6)
Lip width	6.0	6.2 ± 0.3 (5.5–6.5)	6.5 ± 0.4 (6.0–7.0)	6.5, 7.0	6.5 ± 0.5 (6.0–7.0)
Median bulb length	21.0	20.7 ± 2.0 (18.0–24.0)	-	28.0	-
Median bulb width	11.0	10.0 ± 1.0 (9.0–12.0)	-	12.0	-
Anterior end to center median bulb	60.0	56.4 ± 3.5 (51–65)	55.0 ± 0.8 (54.0–56.0)	55.0, 56.0	58.3 ± 1.5 (57.0–60.0)
MB	62.5	55.2 ± 2.3 (51.2–59.0)	54.7 ± 2.6 (53.3–58.5)	53.3, 53.4	55.4 ± 1.7 (54.3–57.4)
Nerve ring to anterior end	72.0	76.7 ± 6.4 (68.0–92.0)	70.5 ± 1.3 (69.0–72.0)	71.0, 72.0	76.0 ± 7.0 (71.0–84.0)
Excretory pore to anterior end	86.0	91.1 ± 11.0 (79.0–118.0)	87.0 ± 2.6 (84.0–90.0)	86.0, 90.0	91.3 ± 8.4 (86.0–101.0)
Pharynx length	96.0	101.5 ± 9.8 (89.0–127.0)	100.8 ± 4.8 (94.0–105.0)	103.0, 105.0	105.3 ± 4.5 (101.1–110.0)
Maximum body diam.	17.0	19.7 ± 3.0 (14.0–24.0)	18.8 ± 1.0 (18.0–20.0)	18.0, 20.5	18.7 ± 2.1 (17.0–21.0)
Tail length	30.0	32.0 ± 5.3 (27.0–47.0)	28.3 ± 1.0 (27.0–29.0)	28.5, 29.0	31.0 ± 1.7 (29.0–32.0)
Anal body diam.	10.0	10.8 ± 1.5 (9.5–15.0)	9.5 ± 0.7 (9.0–10.5)	10.0, 10.5	10.3 ± 0.6 (10.0–11.0)

* Abbreviations: a = body length/greatest body diameter; b = body length/distance from anterior end to pharyngo-intestinal junction; DGO = distance between stylet base and orifice of dorsal pharyngeal gland; c = body length/tail length; c’ = tail length/tail diameter at anus or cloaca; G1 = anterior genital branch length expressed as percentage (%) of the body length; L = overall body length; m = length of conus as percentage of total stylet length; MB = distance between anterior end of body and center of median pharyngeal bulb expressed as percentage (%) of the pharynx length; n = number of specimens on which measurements are based; O = DGO as percentage of stylet length; V = distance from body anterior end to vulva expressed as percentage (%) of the body length.

**Table 4:** Morphometrics of *Paratylenchus pedrami* sp. nov. paratype females, males and other population from Spain. All measurements are in µm and in the form: mean ± s.d. (range).

Measurements and Ratios	Holotype Female	Paratype Females	Paratype Males	Females
Sample code	PR_14	PR_14	PR_14	PR_17
Locality	Córdoba, Córdoba	Córdoba, Córdoba	Córdoba, Córdoba	Córdoba, Córdoba
n	1	20	3	4
L	295	297 ± 30.0 (231–374)	269.7 ± 21.6 (245–285)	305 ± 7.7 (294–312)
a *	21.1	18.6 ± 3.1 (13.7–23.0)	22.6 ± 1.7 (21.1–24.5)	20.1 ± 1.3 (18.9–22.0)
b	3.7	3.7 ± 0.3 (3.2–4.5)	4.5 ± 0.4 (4.0–4.8)	3.6 ± 0.1 (3.5–3.7)
c	13.1	13.0 ± 1.9 (10.0–16.5)	24.2 ± 1.8 (22.3–25.9)	8.6 ± 0.6 (8.2–9.6)
c’	2.6	2.8 ± 0.1 (2.6–3.1)	1.4 ± 0.1 (1.3–1.6)	3.5 ± 0.1 (3.4–3.7)
V or T	81.4	79.9 ± 1.6 (76.0–82.0)	34.7 ± 4.6 (30.1–39.3)	81.2 ± 1.0 (80.1–82.3)
G1	34.9	35.8 ± 6.1 (26.2–50.0)	-	34.4 ± 7.8 (28.8–39.9)
Stylet length	28.0	27.8 ± 1.1 (26.0–30.0)	-	30.9 ± 1.0 (29.5–32.0)
Conus length	22.0	18.5 ± 1.2 (16.0–20.5)	-	22.0 ± 1.6 (20.0–24.0)
m	78.6	66.4 ± 2.8 (61.5–70.4)	-	71.2 ± 3.0 (67.8–75.0)
DGO	4.0	3.9 ± 0.3 (3.5–5.0)	-	4.6 ± 0.5 (4.0–5.0)
O	14.3	14.2 ± 1.4 (12.3–19.2)	-	15.0 ± 1.7 (12.5–16.1)
Lip width	4.5	3.8 ± 0.5 (3.0–5.0)	3.0 ± 0.0	5.5 ± 0.4 (5.0–6.0)
Median bulb length	16.5	19.2 ± 1.4 (17.0–22.0)	-	19.4 ± 1.3 (18.0–21.0)
Median bulb width	7.0	8.5 ± 0.4 (8.0–9.0)	-	8.1 ± 0.9 (7.5–9.5)
Anterior end to center median bulb	45.0	46.0 ± 2.8 (39.0–50.0)	-	49.0 ± 0.0
MB	57.0	57.0 ± 2.9 (53.2–65.3)	-	57.8 ± 0.3 (57.6–58.3)
Nerve ring to anterior end	60.0	59.6 ± 3.5 (53.0–65.0)	-	66.3 ± 1.5 (65.0–68.0)
Excretory pore to anterior end	69.0	71.6 ± 5.3 (62.0–80.0)	65.5	77.8 ± 3.5 (74.0–82.0)
Pharynx length	79.0	80.1 ± 4.6 (70.0–86.0)	60.3 ± 9.7 (52.0–71.0)	84.8 ± 0.5 (84.0–85.0)
Maximum body diam.	14.0	16.5 ± 3.3 (12.0–23.0)	12.0 ± 1.8 (10.0–13.5)	15.3 ± 1.0 (14.0–16.5)
Tail length	22.5	23.2 ± 3.1 (18.0–28.0)	11.2 ± 1.3 (10.0–12.5)	35.5 ± 2.6 (32.0–38.0)
Anal body diam.	8.5	8.3 ± 0.9 (7.0–9.5)	7.8 ± 1.0 (7.0–9.0)	10.1 ± 0.6 (9.5–11.0)
Spicules	-	-	16.2 ± 2.3 (14.0–18.5)	-
Gubernaculum	-	-	3.5 ± 0.5 (3.0–4.0)	-

* Abbreviations: a = body length/greatest body diameter; b = body length/distance from anterior end to pharyngo-intestinal junction; DGO = distance between stylet base and orifice of dorsal pharyngeal gland; c = body length/tail length; c’ = tail length/tail diameter at anus or cloaca; G1 = anterior genital branch length expressed as percentage (%) of the body length; L = overall body length; m = length of conus as percentage of total stylet length; MB = distance between anterior end of body and center of median pharyngeal bulb expressed as percentage (%) of the pharynx length; n = number of specimens on which measurements are based; O = DGO as percentage of stylet length; T = distance from cloacal aperture to anterior end of testis expressed as percentage (%) of the body length; V = distance from body anterior end to vulva expressed as percentage (%) of the body length.

**Table 5:** Morphometrics of *Paratylenchus zurgenerus* sp. nov. paratype females from Zurgena, Almería province (Spain). All measurements are in µm and in the form: mean ± s.d. (range).

Measurements and Ratios	Holotype Female	Paratype Females
Sample code	PR_111	PR_111
n	1	19
L	370	356.9 ± 32.1 (316–418)
a *	20.0	20.1 ± 1.7 (17.5–23.7)
b	3.7	3.9 ± 0.4 (3.4–4.9)
c	11.9	13.8 ± 1.7 (11.0–17.0)
c’	2.8	2.7 ± 0.3 (2.0–3.2)
V	83.2	84.8 ± 1.0 (83.3–86.6)
G1	40.0	28.4 ± 6.9 (18.2–43.5)
Stylet length	15.0	15.4 ± 0.6 (14.0–16.0)
Conus length	9.0	9.0 ± 0.6 (8.0–10.0)
m	60.0	58.6 ± 3.4 (53.1–66.7)
DGO	5.5	5.0 ± 0.8 (3.5–6.0)
O	36.7	32.4 ± 4.4 (22.6–37.5)
Lip width	6.0	6.7 ± 0.6 (6.0–8.0)
Median bulb length	-	22.1 ± 1.96 (19.0–26.0)
Median bulb width	-	10.0 ± 0.9 (9.0–12.0)
Anterior end to center median bulb	55.0	47.7 ± 3.0 (41.0–52.0)
MB	55.3	51.6 ± 2.1 (47.2–55.1)
Nerve ring to anterior end	70.0	64.9 ± 4.2 (59.0–73.0)
Excretory pore to anterior end	83.0	81.3 ± 7.4 (67.0–94.0)
Pharynx length	99.5	92.5 ± 4.5 (85.0–101.0)
Maximum body diam.	18.5	17.8 ± 2.0 (15.5–22.0)
Tail length	31.0	26.2 ± 3.5 (22.0–34.5)
Anal body diam.	11.0	9.7 ± 1.1 (8.0–11.5)

* Abbreviations: a = body length/greatest body diameter; b = body length/distance from anterior end to pharyngo-intestinal junction; DGO = distance between stylet base and orifice of dorsal pharyngeal gland; c = body length/tail length; c’ = tail length/tail diameter at anus or cloaca; G1 = anterior genital branch length expressed as percentage (%) of the body length; L = overall body length; m = length of conus as percentage of total stylet length; MB = distance between anterior end of body and center of median pharyngeal bulb expressed as percentage (%) of the pharynx length; n = number of specimens on which measurements are based; O = DGO as percentage of stylet length; V = distance from body anterior end to vulva expressed as percentage (%) of the body length.

**Table 6:** Morphometrics of *Paratylenchus baldaccii* Raski, 1975, *Paratylenchus enigmaticus* Munawar, Yevtushenko, Palomares-Rius & Castillo, 2021, *Paratylenchus goodeyi* (Oostenbrink, 1953) Raski, 1962, *Paratylenchus holdemani* Raski, 1975 and *Paratylenchus israelensis* (Raski, 1973) Siddiqi, 1986 from several localities in Spain. All measurements are in µm and in the form: mean ± s.d. (range).

Measurements and Ratios	*P. baldaccii*	*P. enigmaticus*	*P. goodeyi*	*P. israelensis*			
	Females	Females	Females	Females	Males	Females	Females
Sample code	PR_152	PR_193	PR_14	PR_82	PR_82	PR_79	PR_11
Locality	Cantillana, Sevilla	La Almunia, Zaragoza	Córdoba, Córdoba	Martos, Jaén	Martos, Jaén	Valenzuela, Córdoba	Córdoba, Córdoba
n	11	5	8	7	4	7	3
L	309.2 ± 25.0 (272–354)	364.6 ± 23.5 (324–383)	411.1 ± 10.5 (396–427)	396.4 ± 36.5 (345–441)	420.3 ± 25 (388–441)	431.6 ± 26.9 (400–464)	472.7 ± 27.0 (446–500)
a *	20.5 ± 2.7 (14.7–25.3)	19.9 ± 1.5 (17.6–21.6)	22.8 ± 2.6 (19.9–26.8)	25.1 ± 1.4 (23.5–27.2)	30.5 ± 3.6 (27.5–35.1)	21.0 ± 2.4 (18.0–23.6)	25.2 ± 1.4 (23.6–26.2)
b	3.8 ± 0.4 (3.4–4.4)	3.8 ± 0.2 (3.6–4.2)	3.9 ± 0.2 (3.5–4.2)	3.9 ± 0.3 (3.5–4.3)	4.9 ± 0.24 (4.5–5.1)	4.1 ± 0.4 (3.8–4.8)	4.2 ± 0.3 (3.9–4.5)
c	10.0 ± 1.9 (8.0–14.8)	14.6 ± 1.6 (12.0–16.0)	11.7 ± 0.6 (10.7–12.2)	13.8 ± 1.1 (12.2–15.0)	11.4 ± 1.1 (10.5–13.0)	11.8 ± 1.6 (9.2–14.4)	10.5 ± 1.1 (9.4–11.6)
c’	3.5 ± 0.1 (3.4–3.7)	2.7 ± 0.1 (2.5–2.8)	3.6 ± 0.2 (3.2–3.8)	3.0 ± 0.4 (2.6–3.5)	3.5 ± 0.22 (3.4–3.9)	3.4 ± 0.4 (2.9–4.0)	4.0 ± 0.6 (3.3–4.5)
V or T	79.8 ± 1.0 (77.5–81.2)	83.4 ± 1.3 (81.2–84.4)	81.7 ± 0.5 (81.0–82.4)	81.5 ± 1.1 (79.9–83.4)	47.5 ± 4.6 (44.8–52.8)	80.3 ± 1.0 (78.7–81.8)	78.0 ± 2.0 (76.1–80.0)
G1	32.7 ± 4.9 (27.0–38.9)	33.7 ± 7.8 (23.1–42.9)	30.8 ± 1.9 (29.2–33.9)	35.9 ± 10.2 (28.7–43.1)	-	33.0 ± 2.7 (30.0–37.1)	33.8 ± 3.9 (29.4–36.4)
Stylet length	30.5 ± 1.1 (29.0–33.0)	27.6 ± 0.9 (27.0–29.0)	49.6 ± 2.6 (46.0–53.0)	26.7 ± 1.5 (24.0–29.0)	14.5 ± 0.6 (14.0–15.0)	26.0 ± 1.2 (25.0–28.0)	26.5 ± 0.5 (26.0–27.0)
Conus length	21.2 ± 1.5 (19.0–24.0)	17.5 ± 0.9 (17.0–19.0)	40.2 ± 2.6 (37.0–44.0)	16.5 ± 1.6 (14.5–19.0)	8.3 ± 0.4 (8.0–8.8)	16.6 ± 0.7 (15.5–17.5)	16.5 ± 0.5 (16.0–17.0)
m	69.3 ± 2.9 (63.3–72.7)	63.4 ± 1.2 (62.5–65.5)	80.9 ± 1.4 (79.2–83.0)	61.7 ± 3.2 (57.7–66.7)	57.5 ± 3.1 (53.3–60.7)	64.1 ± 2.2 (62.0–68.0)	62.3 ± 0.7 (61.5–63.0)
DGO	5.5 ± 0.7 (4.5–6.5)	5.9 ± 1.6 (4.0–7.5)	5.3 ± 0.4 (4.5–5.5)	6.4 ± 0.5 (5.5–7.0)	5.3 ± 0.6 (5.0–6.0)	6.6 ± 0.7 (6.0–7.5)	5.8 ± 0.3 (5.5–6.0)
O	17.9 ± 2.5 (15.2–22.4)	21.5 ± 6.0 (14.8–27.8)	10.6 ± 1.1 (8.8–12.0)	24.1 ± 2.0 (21.2–27.1)	36.3 ± 3.4 (33.3–40.0)	25.3 ± 2.4 (22.2–28.3)	22.0 ± 1.2 (20.8–23.1)
Lip width	5.5 ± 0.5 (4.5–6.0)	7.6 ± 0.4 (7.0–8.0)	5.1 ± 0.2 (5.0–5.5)	6.7 ± 0.6 (6.0–7.5)	3.4 ± 0.5 (3.0–4.0)	8.8 ± 0.3 (8.5–9.0)	8.3 ± 0.8 (7.5–9.0)
Median bulb length	19.7 ± 2.5 (17.0–24.0)	25.0 ± 1.0 (24.0–26.0)	20.6 ± 2.1 (17.0–23.0)	20.4 ± 2.8 (16.5–24.0)	16.7 ± 0.6 (16.0–17.0)	25.8 ± 3.1 (23.0–31.0)	24.3 ± 2.1 (22.0–26.0)
Median bulb width	8.6 ± 0.7 (7.5–9.5)	11.2 ± 0.8 (10.5–12.0)	10.9 ± 0.3 (10.5–11.5)	9.8 ± 0.6 (9.0–10.5)	7.8 ± 0.3 (7.5–8.0)	11.9 ± 1.2 (10.5–14.0)	8.5 ± 0.5 (8.0–9.0)
Anterior end to center median bulb	47.6 ± 3.1 (43.0–52.0)	53.6 ± 3.0 (50.0–57.0)	73.7 ± 2.8 (70.0–77.0)	57.1 ± 3.0 (52.5–60.5)	52.6 ± 5.1 (45.0–56.0)	60.2 ± 3.8 (55.0–65.0)	60.7 ± 1.5 (59.0–62.0)
MB	59.0 ± 1.2 (57.6–60.5)	55.8 ± 1.0 (54.4–56.8)	68.5 ± 3.4 (62.6–72.8)	56.4 ± 0.9 (55.2–57.6)	-	56.8 ± 1.8 (54.7–60.0)	54.2 ± 0.9 (53.2–54.9)
Nerve ring to anterior end	62.1 ± 4.0 (57.0–68.0)	70.4 ± 4.0 (66.0–75.0)	87.5 ± 7.4 (78.0–96.0)	75.5 ± 5.3 (68.0–85.5)	62.3 ± 5.3 (58.5–66.0)	78.4 ± 5.9 (71.0–89.5)	78.3 ± 3.2 (76.0–82.0)
Excretory pore to anterior end	75.3 ± 6.5 (69.0–90.0)	87.6 ± 5.7 (81.0–94.0)	91.6 ± 6.8 (82.0–102.0)	87.2 ± 6.5 (79.5–96.5)	79.5 ± 6.1 (72.5–84.0)	90.2 ± 5.4 (84.0–97.0)	92.0 ± 1.0 (91.0–93.0)
Pharynx length	82.3 ± 4.5 (75.0–87.0)	96.2 ± 6.5 (88.0–103.0)	107 ± 8.3 (97.0–123.0)	100.7 ± 4.6 (93.0–107.0)	86.3 ± 5.9 (78.0–91.0)	106.1 ± 8.3 (95.0–117.0)	112.0 ± 1.0 (111–113)
Maximum body diam.	15.3 ± 1.6 (12.5–18.5)	18.4 ± 1.7 (16.5–21.0)	18.3 ± 2.2 (15.0–20.5)	15.8 ± 0.8 (14.5–16.5)	13.9 ± 1.0 (12.5–15.0)	20.7 ± 2.4 (18.0–23.5)	18.8 ± 1.6 (17.0–20.0)
Tail length	31.7 ± 5.2 (24.0–40.0)	25.0 ± 1.4 (23.5–27.0)	35.3 ± 1.5 (33.5–37.5)	228.9 ± 3.9 (23.0–33.5)	37.1 ± 2.7 (34.0–40.5)	36.9 ± 5.0 (32.0–46.0)	45.3 ± 4.0 (43.0–50.0)
Anal body diam.	9.0 ± 1.2 (7.0–11.0)	9.4 ± 0.4 (9.0–10.0)	9.9 ± 0.6 (9.0–10.5)	9.8 ± 0.5 (9.0–10.5)	10.5 ± 0.4 (44.8–52.8)	11.0 ± 1.1 (10.0–12.5)	11.5 ± 1.3 (10.5–13.0)
Spicules	-	-	-	-	21.8 ± 0.96 (21.0–23.0)	-	-
Gubernaculum	-	-	-	-	5.1 ± 0.25 (5.0–5.5)	-	-

* Abbreviations: a = body length/greatest body diameter; b = body length/distance from anterior end to pharyngo-intestinal junction; DGO = distance between stylet base and orifice of dorsal pharyngeal gland; c = body length/tail length; c’ = tail length/tail diameter at anus or cloaca; G1 = anterior genital branch length expressed as percentage (%) of the body length; L = overall body length; m = length of conus as percentage of total stylet length; MB = distance between anterior end of body and center of median pharyngeal bulb expressed as percentage (%) of the pharynx length; n = number of specimens on which measurements are based; O = DGO as percentage of stylet length; T = distance from cloacal aperture to anterior end of testis expressed as percentage (%) of the body length; V = distance from body anterior end to vulva expressed as percentage (%) of the body length.

**Table 7:** Morphometrics of *Paratylenchus hamatus* Thorne & Allen, 1950 from several localities in Spain. All measurements are in µm and in the form: mean ± s.d. (range).

Measurements and Ratios	Females	Females	Females	Females	Males
Sample code	PR_44	PR_115	PR_207	PR_187	PR_187
Locality	Gibraleón, Huelva	Lúcar, Almería	Sástago, Zaragoza	Ariza, Zaragoza	Ariza, Zaragoza
n	4	4	5	15	2
L	362 ± 11.0 (347–373)	386.5 ± 28.8 (355–420)	421.6 ± 46.9 (377–487)	373.5 ± 31.4 (327–433.5)	744, 789
a *	22.2 ± 1.4 (20.4–23.4)	20.7 ± 1.8 (19.2–23.3)	20.5 ± 3.7 (16.5–24.9)	20.2 ± 3.1 (13.5–25.3)	40.5, 45.1
b	4.0 ± 0.2 (3.9–4.2)	3.8 ± 0.3 (3.4–4.1)	4.6 ± 0.4 (4.3–5.2)	3.8 ± 0.2 (3.5–4.4)	4.7, 6.7
c	12.9 ± 0.8 (11.7–13.6)	13.9 ± 1.7 (11.7–15.4)	13.6 ± 1.8 (11.5–16.2)	12.6 ± 1.0 (10.4 ± 14.6)	17.5, 18.6
c’	3.3 ± 0.1 (3.2–3.4)	3.0 ± 0.3 (2.6–3.3)	3.1 ± 0.3 (2.9–3.6)	2.9 ± 0.3 (2.3–3.6)	3.5, 4.3
V or T	81.7 ± 0.9 (80.4 ± 82.6)	81.9 ± 0.4 (81.5–82.2)	81.8 ± 1.3 (80.4–83.6)	82.0 ± 1.1 (80.1–84.2)	53.0, 59.1
G1	41.7 ± 6.9 (33.3–47.7)	39.7 ± 4.9 (35.6–45.6)	55.9 ± 3.2 (52.3–59.9)	46.5 ± 7.6 (33.0–53.5)	-
Stylet length	31.1 ± 1.9 (29.0–33.0)	33.4 ± 2.1 (31.5–36.0)	30.1 ± 0.7 (29.0–31.0)	30.9 ± 1.1 (28.0–32.5)	16.5, 19.5
Conus length	20.8 ± 1.8 (19.0–23.0)	22.5 ± 1.7 (21.0–25.0)	20.2 ± 2.2 (18.0–23.0)	19.8 ± 1.1 (18.0–21.5)	8.0, 8.0
m	66.6 ± 2.8 (65.0–70.8)	67.4 ± 2.6 (64.7–69.8)	67.0 ± 5.7 (62.1–74.2)	59.8 ± 2.8 (60.0–69.4)	41.0, 48.5
DGO	3.8 ± 0.3 (3.5–4.0)	7.1 ± 0.9 (6.0–8.0)	6.5 ± 0.8 (5.5–7.5)	-	4.5, 5.5
O	12.0 ± 0.3 (11.7–12.3)	21.4 ± 3.0 (18.8–25.4)	21.6 ± 2.4 (18.3–24.2)	-	27.3, 28.2
Lip width	4.5 ± 0.4 (4.0–5.0)	6.3 ± 0.3 (6.0–6.5)	6.4 ± 0.4 (6.0–7.0)	6.2 ± 0.4 (5.5–7.0)	6.5, 7.5
Median bulb length	-	22.8 ± 2.9 (21.0–27.0)	26.8 ± 1.0 (26.0–28.0)	17.9 ± 2.0 (13.5–20.0)	10.5, 12.5
Median bulb width	-	9.4 ± 1.9 (7.0–11.5)	11.0 ± 0.8 (10.0–12.0)	9.4 ± 1.4 (7.0–12.0)	8.0, 10.0
Anterior end to center median bulb	52.5 ± 1.7 (50.0–54.0)	56.0 ± 1.8 (54.0–58.0)	55.0 ± 2.0 (53.0–58.0)	56.1 ± 3.7 (51.0–64.0)	64.5, 70.0
MB	58.0 ± 2.2 (55.6–60.2)	55.4 ± 2.1 (52.9–58.1)	60.5 ± 3.6 (56.8–65.1)	57.3 ± 2.6 (53.2–62.2)	44.6, 55.1
Nerve ring to anterior end	71.3 ± 5.4 (67.0–79.0)	74.3 ± 6.9 (67.0–83.0)	66.0 ± 1.7 (63.0–67.0)	-	85.5, 87.0
Excretory pore to anterior end	82.0 ± 5.2 (76.0–88.0)	85.9 ± 7.1 (78.5–92.0)	83.8 ± 2.3 (81.0–87.0)	87.8 ± 6.7 (77.5–100.0)	104.0, 108.0
Pharynx length	90.5 ± 3.1 (88.0–95.0)	101.3 ± 5.6 (93.0–105.0)	91.0 ± 3.8 (86.0–95.0)	98.3 ± 6.3 (85.5–109.0)	117.0, 157.0
Maximum body diam.	16.4 ± 0.8 (15.5–17.0)	18.8 ± 2.3 (16.0–21.5)	21.2 ± 4.9 (18.0–29.0)	19.0 ± 3.7 (17.0–27.5)	16.5, 19.5
Tail length	28.3 ± 2.2 (26.0–31.0)	28.4 ± 5.5 (23.0–36.0)	31.2 ± 3.7 (26.0–36.0)	29.7 ± 2.0 (25.5–33.0)	40.0, 45.0
Anal body diam.	8.6 ± 0.5 (8.0–9.0)	9.5 ± 1.1 (8.5–11.0)	10.0 ± 0.6 (9.0–10.5)	10.2 ± 1.2 (8.0–12.5)	10.5, 11.5
Spicules	-	-	-	-	22.0, 23.5
Gubernaculum	-	-	-	-	11.0, 13.5

* Abbreviations: a = body length/greatest body diameter; b = body length/distance from anterior end to pharyngo-intestinal junction; DGO = distance between stylet base and orifice of dorsal pharyngeal gland; c = body length/tail length; c’ = tail length/tail diameter at anus or cloaca; G1 = anterior genital branch length expressed as percentage (%) of the body length; L = overall body length; m = length of conus as percentage of total stylet length; MB = distance between anterior end of body and center of median pharyngeal bulb expressed as percentage (%) of the pharynx length; n = number of specimens on which measurements are based; O = DGO as percentage of stylet length; T = distance from cloacal aperture to anterior end of testis expressed as percentage (%) of the body length; V = distance from body anterior end to vulva expressed as percentage (%) of the body length.

**Table 8:** Morphometrics of *Paratylenchus tenuicaudatus* Wu, 1961 from several localities in Spain. All measurements are in µm and in the form: mean ± s.d. (range).

Measurements and Ratios	Females	Males	Females	Females	Females
Sample code	PR_124	PR_124	PR_129	PR_168	PR_208
Locality	Caravaca, Murcia	Caravaca, Murcia	Calasparra, Murcia	Sollana, Valencia	Sástago, Zaragoza
n	3	2	9	4	3
L	368.7 ± 20.4 (354–392)	356, 364	376.6 ± 31.0 (307–407)	358.5 ± 44.7 (292–389)	405.3 ± 10.3 (394–414)
a *	23.5 ± 1.3 (22.1–24.5)	29.1, 29.7	22.7 ± 3.7 (16.5–28.4)	23.6 ± 2.0 (20.9–25.1)	18.3 ± 0.8 (17.4–18.8)
b	4.5 ± 0.2 (4.2–4.6)	4.4, 4.7	3.8 ± 0.3 (3.4–4.2)	4.0 ± 0.4 (3.4–4.2)	4.0 ± 0.0
c	11.7 ± 1.9 (10.4–13.8)	11.1, 12.3	11.7 ± 1.3 (10.5–14.4)	9.7 ± 0.9 (8.7–10.5)	10.3 ± 0.5 (10.0–10.9)
c’	3.7 ± 0.4 (3.3–4.0)	3.0, 3.8	3.6 ± 0.5 (2.8–4.8)	3.8 ± 0.5 (3.3–4.2)	3.4 ± 0.1 (3.3–3.5)
V or T	83.0 ± 1.5 (82.1–84.7)	39.3, 46.3	80.8 ± 1.3 (78.3–82.5)	80.6 ± 0.8 (79.6–81.5)	80.2 ± 0.8 (79.2–80.7)
G1	46.3 ± 10.8 (33.9–52.8)	-	41.3 ± 7.4 (27.6–50.4)	34.8 ± 5.5 (29.8–41.2)	43.7 ± 3.3 (40.1–46.6)
Stylet length	29.3 ± 0.6 (29.0–30.0)	-	30.7 ± 1.1 (29.0–32.0)	30.5 ± 1.5 (29.0–32.0)	32.7 ± 0.8 (32.0–33.5)
Conus length	17.7 ± 0.6 (17.0–18.0)	-	21.0 ± 1.2 (19.5–23.0)	20.5 ± 1.9 (18.0–22.0)	21.7 ± 1.2 (21.0–23.0)
m	60.9 ± 2.0 (58.6–62.1)	-	68.4 ± 1.7 (66.7–71.9)	67.1 ± 4.1 (61.0–69.8)	66.3 ± 2.1 (64.6–68.7)
DGO	5.3 ± 0.6 (5.0–6.0)	-	5.0 ± 0.9 (4.0–6.5)	6.7 ± 0.6 (6.0–7.0)	7.2 ± 0.3 (7.0–7.5)
O	18.4 ± 2.0 (17.2–20.7)	-	16.3 ± 2.7 (12.7–20.3)	22.2 ± 1.5 (20.7–23.7)	21.9 ± 0.4 (21.5–22.4)
Lip width	6.2 ± 0.3 (6.0–6.5)	4.0	4.9 ± 0.8 (4.0–6.5)	5.3 ± 0.5 (5.0–6.0)	7.2 ± 0.3 (7.0–7.5)
Median bulb length	-	-	23.4 ± 2.9 (18.0–27.0)	17.5 ± 2.1 (16.0–19.0)	28.0 ± 1.0 (27.0–29.0)
Median bulb width	-	-	9.1 ± 1.4 (7.0–11.5)	8.8 ± 1.3 (7.5–10.0)	12.8 ± 0.8 (12.0–13.5)
Anterior end to center median bulb	44.7 ± 1.2 (44.0–46.0)	44.0, 46.0	57.9 ± 3.6 (52.0–63.0)	50.8 ± 3.2 (49.0–54.5)	61.0 ± 1.0 (60.0–62.0)
MB	54.3 ± 2.4 (51.8–56.4)	56.1, 58.7	57.7 ± 3.4 (54.4–64.2)	56.2 ± 3.2 (52.7–58.9)	60.4 ± 0.2 (60.2–60.6)
Nerve ring to anterior end	61.7 ± 4.5 (57.0–66.0)	54.0, 55.0	73.9 ± 5.3 (66.5–81.0)	64.0 ± 6.2 (57.0–69.0)	80.3 ± 0.8 (79.2–80.7)
Excretory pore to anterior end	75.3 ± 2.3 (74.0–78.0)	59.0, 71.0	80.4 ± 7.7 (68.0–88.0)	75.2 ± 2.4 (72.5–77.0)	93.3 ± 7.2 (85.0–98.0)
Pharynx length	82.3 ± 3.8 (78.0–85.0)	75.0, 82.0	98.1 ± 7.6 (88.0–110.0)	90.4 ± 3.2 (86.0–93.0)	101.0 ± 2.0 (99.0–103.0)
Maximum body diam.	15.7 ± 0.6 (15.0–16.0)	12.0, 12.5	17.1 ± 3.8 (12.0–24.0)	15.1 ± 0.9 (14.0–16.0)	22.2 ± 1.3 (21.0–23.5)
Tail length	32.0 ± 5.3 (26.0–36.0)	29.5, 32.0	32.6 ± 4.8 (25.0–38.0)	37.3 ± 6.9 (28.0–43.0)	39.3 ± 1.5 (38.0–41.0)
Anal body diam.	8.7 ± 0.6 (8.0–9.0)	8.5, 10.0	9.0 ± 1.2 (7.0–11.0)	9.8 ± 1.7 (8.5–12.0)	11.7 ± 0.6 (11.0–12.0)
Spicules	-	22.0, 25.0	-	-	-
Gubernaculum	-	6.0, 7.5	-	-	-

* Abbreviations: a = body length/greatest body diameter; b = body length/distance from anterior end to pharyngo-intestinal junction; DGO = distance between stylet base and orifice of dorsal pharyngeal gland; c = body length/tail length; c’ = tail length/tail diameter at anus or cloaca; G1 = anterior genital branch length expressed as percentage (%) of the body length; L = overall body length; m = length of conus as percentage of total stylet length; MB = distance between anterior end of body and center of median pharyngeal bulb expressed as percentage (%) of the pharynx length; n = number of specimens on which measurements are based; O = DGO as percentage of stylet length; T = distance from cloacal aperture to anterior end of testis expressed as percentage (%) of the body length; V = distance from body anterior end to vulva expressed as percentage (%) of the body length.

**Table 9:** Morphometrics of *Paratylenchus veruculatus* Wu, 1962 from several localities in Spain. All measurements are in µm and in the form: mean ± s.d. (range).

Measurements and Ratios	Females	Females	Females	Females	Females
Sample code	PR_122	PR_106	PR_115	PR_118	PR_193
Locality	Puebla de Don Fadrique, Granada	Sta. Mª Nieva, Almería	Lúcar, Almería	Serón, Almería	La Almunia, Zaragoza
n	10	4	10	4	10
L	376.1 ± 30.4 (354–436)	381.5 ± 25.4 (349–407)	374.5 ± 37.8 (303–445)	379.5 ± 19.2 (353–395)	363.8 ± 45.8 (279–441)
a *	20.4 ± 2.5 (17.0–24.3)	20.7 ± 0.3 (17.5–24.7)	20.4 ± 1.9 (16.8–23.9)	22.2 ± 0.7 (21.4–23.1)	19.8 ± 1.6 (16.4–21.7)
b	4.0 ± 0.2 (3.7–4.4)	4.2 ± 0.4 (3.8–4.8)	4.0 ± 0.4 (3.4–4.7)	4.1 ± 0.3 (3.8–4.4)	3.8 ± 0.3 (3.4–4.4)
c	14.8 ± 2.0 (11.8–19.7)	12.9 ± 0.6 (12.0–13.4)	15.5 ± 1.7 (13.1–18.4)	13.7 ± 0.3 (13.5–14.1)	14.0–1.5 (11.2–15.8)
c’	2.5 ± 0.2 (2.3–2.8)	2.7 ± 0.3 (2.3–2.9)	2.4 ± 0.3 (2.1–2.8)	2.8 ± 0.04 (2.7–2.8)	2.8 ± 0.2 (2.5–3.0)
V	84.2 ± 1.0 (82.7–85.5)	84.0 ± 0.9 (83.1–84.8)	83.6 ± 0.6 (82.8–84.5)	83.8 ± 1.1 (82.8–84.9)	84.3 ± 0.8 (83.1–85.4)
G1	36.5 ± 7.3 (22.3–47.7)	34.2 ± 12.5 (26.7–52.9)	38.5 ± 4.1 (32.0–43.2)	33.6 ± 0.8 (25.9–43.6)	35.6 ± 4.7 (29.8–44.4)
Stylet length	15.1 ± 0.6 (14.0–16.0)	15.9 ± 0.3 (15.5–16.0)	15.5 ± 0.6 (14.5–16.5)	15.6 ± 0.25 (15.5–16.0)	15.7 ± 0.7 (15.0–17.0)
Conus length	9.1 ± 0.7 (7.5–10.0)	9.9 ± 0.3 (9.5–10.0)	9.5 ± 0.6 (8.0–10.0)	9.8 ± 0.3 (9.5–10.0)	9.6 ± 0.5 (9.0–10.5)
m	60.4 ± 5.5 (50.0–66.7)	62.2 ± 0.6 (61.3–62.5)	60.9 ± 2.3 (55.2–63.3)	61.4 ± 2.5 (59.4–64.5)	61.3 ± 1.8 (58.1–63.3)
DGO	5.2 ± 1.2 (3.0–7.0)	4.3 ± 0.3 (4.0–4.5)	4.5 ± 0.6 (3.5–5.5)	3.6 ± 0.6 (3.0–4.5)	4.5 ± 0.4 (4.0–5.0)
O	34.3 ± 7.4 (20.0–46.7)	26.8 ± 2.1 (25.0–29.0)	28.7 ± 3.3 (21.9–33.3)	22.8 ± 3.9 (18.8–28.1)	28.8 ± 2.7 (25.0–33.3)
Lip width	6.6 ± 0.6 (6.0–7.5)	6.0 ± 0.4 (5.5–6.5)	6.3 ± 0.4 (6.0–7.0)	7.0 ± 0.4 (6.5–7.5)	6.7 ± 0.4 (6.0–7.5)
Median bulb length	23.8 ± 2.4 (22.0–27.0)	25.3 ± 1.0 (24.0–26.0)	24.6 ± 5.0 (19.0–33.0)	26.5 ± 0.7 (26.0–27.0)	20.8 ± 1.5 (19.0–22.0)
Median bulb width	8.1 ± 0.6 (7.5–9.0)	8.4 ± 0.5 (8.0–9.0)	9.3 ± 1.3 (8.0–12.0)	9.5 ± 0.7 (9.0–10.0)	9.3 ± 1.2 (8.0–11.0)
Anterior end to center median bulb	49.3 ± 2.8 (44.0–53.0)	47.5 ± 2.4 (45.0–50.0)	48.0 ± 2.1 (45.0–51.0)	49.8 ± 2.1 (47.0–52.0)	49.0 ± 3.4 (43.0–54.0)
MB	51.2 ± 2.2 (47.8–54.1)	52.4 ± 1.3 (51.1–53.8)	51.6 ± 1.5 (48.4–53.4)	53.2 ± 3.8 (50.5–58.8)	50.0 ± 2.7 (46.7–53.8)
Nerve ring to anterior end	68.6 ± 6.5 (60.0–80.0)	65.0 ± 4.4 (60.0–70.0)	65.1 ± 4.1 (59.0–70.0)	69.5 ± 4.4 (64.0–74.0)	68.3 ± 5.3 (61.0–76.0)
Excretory pore to anterior end	82.6 ± 8.1 (69.0–97.0)	85.5 ± 6.6 (79.0–93.0)	81.0 ± 6.3 (70.0–92.0)	84.3 ± 6.7 (75.0–90.0)	84.0 ± 7.3 (75.0–97.0)
Pharynx length	94.8 ± 7.1 (84.0–108.0)	90.8 ± 4.9 (85.0–97.0)	93.0 ± 3.8 (88.0–101.0)	94.0 ± 9.4 (80.0–100.0)	95.4 ± 7.0 (81.0–105.0)
Maximum body diam.	18.7 ± 2.7 (15.0–23.0)	18.6 ± 1.5 (16.5–20.0)	18.4 ± 1.7 (15.0–20.0)	17.1 ± 0.6 (16.5–18.0)	18.5 ± 2.3 (16.5–24.0)
Tail length	25.9 ± 4.6 (18.0–36.5)	29.6 ± 1.3 (28.5–31.5)	24.5 ± 4.0 (16.5–30.0)	27.6 ± 1.1 (26.0–28.5)	26.1 ± 1.7 (23.0–28.0)
Anal body diam.	10.4 ± 1.4 (8.0–13.0)	11.3 ± 1.6 (10.0–13.5)	10.0 ± 0.9 (8.0–11.0)	10.0 ± 0.4 (9.5–10.5)	9.4 ± 0.8 (8.0–11.0)

* Abbreviations: a = body length/greatest body diameter; b = body length/distance from anterior end to pharyngo-intestinal junction; DGO = distance between stylet base and orifice of dorsal pharyngeal gland; c = body length/tail length; c’ = tail length/tail diameter at anus or cloaca; G1 = anterior genital branch length expressed as percentage (%) of the body length; L = overall body length; m = length of conus as percentage of total stylet length; MB = distance between anterior end of body and center of median pharyngeal bulb expressed as percentage (%) of the pharynx length; n = number of specimens on which measurements are based; O = DGO as percentage of stylet length; T = distance from cloacal aperture to anterior end of testis expressed as percentage (%) of the body length; V = distance from body anterior end to vulva expressed as percentage (%) of the body length.

## Data Availability

The datasets generated during and/or analyzed during the current study are available from the corresponding author on reasonable request.
